# Experimental and Theoretical Study of the Reaction of F_2_ with Thiirane

**DOI:** 10.3390/molecules29153636

**Published:** 2024-07-31

**Authors:** Yuri Bedjanian, Antoine Roose, Valérie Vallet, Manolis N. Romanias

**Affiliations:** 1 Institut de Combustion, Aérothermique, Réactivité et Environnement (ICARE), CNRS, 45071 Orléans, France; 2 IMT Nord Europe, Institut Mines-Télécom, University Lille, Centre for Energy and Environment, 59000 Lille, France; antoine.roose@imt-nord-europe.fr (A.R.); emmanouil.romanias@imt-nord-europe.fr (M.N.R.); 3 University Lille, CNRS, UMR 8523-PhLAM-Physique des Lasers Atomes et Molécules, 59000 Lille, France; valerie.vallet@univ-lille.fr

**Keywords:** fluorine, C_2_H_4_S, gas-phase kinetics, rate constant, Boltzmann population, enthalpy

## Abstract

The kinetics of the F_2_ reaction with thiirane (C_2_H_4_S) was studied for the first time in a flow reactor combined with mass spectrometry at a total helium pressure of 2 Torr and in the temperature range of 220 to 800 K. The rate constant of the title reaction was determined under pseudo-first-order conditions, either monitoring the kinetics of F_2_ or C_2_H_4_S consumption in excess of thiirane or of F_2_, respectively: *k*_1_ = (5.79 ± 0.17) × 10^−12^ exp(−(16 ± 10)/T) cm^3^ molecule^−1^ s^−1^ (the uncertainties represent precision of the fit at the 2*σ* level, with the total 2σ relative uncertainty, including statistical and systematic errors on the rate constant being 15% at all temperatures). HF and CH_2_CHSF were identified as primary products of the title reaction. The yield of HF was measured to be 100% (with an accuracy of 10%) across the entire temperature range of the study. Quantum computations revealed reaction enthalpies ranging from −409.9 to −509.1 kJ mol^−1^ for all the isomers/conformers of the products, indicating a strong exothermicity. Boltzmann relative populations were then established for different temperatures.

## 1. Introduction

The reactivity of F_2_ molecules has certain specific features and is of interest for both experimental and theoretical studies. One notable feature is that molecular fluorine exhibits surprisingly high reactivity towards certain closed-shell molecules. For example, it has been demonstrated that reactions of F_2_ with organosulfur compounds, CH_3_SCH_3_ and CH_3_SSCH_3_, and with limonene are barrierless reactions [1,2,3]. Unexpectedly high rate constants (for reactions between two closed-shell molecules), 1.6 × 10^−11^ at 298 K [4] and 1.9 × 10^−12^ cm^3^ molecule^−1^ s^−1^ at T = 278–360 K [3], were reported for reaction of F_2_ with dimethyl sulfide (CH_3_SCH_3_) and limonene, respectively. The current kinetic and mechanistic database on F_2_ reactions with stable molecules is very limited [5], especially regarding information on reaction products and the temperature dependence of reaction rate constants. To better understand the nature of the specific reactivity of the F_2_ molecule, additional kinetic and mechanistic studies (preferably over a wide temperature range) are necessary.

In the present work, we report the results of combined experimental and theoretical study of the reaction of molecular fluorine with another organosulfur compound, thiirane (C_2_H_4_S), over a wide temperature range (from 220 to 800 K):

F_2_ + C_2_H_4_S → products (1)

The reaction rate constant as well as the reaction products are reported for the first time. To our knowledge, no information exists in the literature regarding the spontaneity and thermodynamical stability of this reaction. Quantum computations have been carried out to determine the thermodynamical parameter of the reaction.

## 2. Results and Discussion

**Figure 1:**
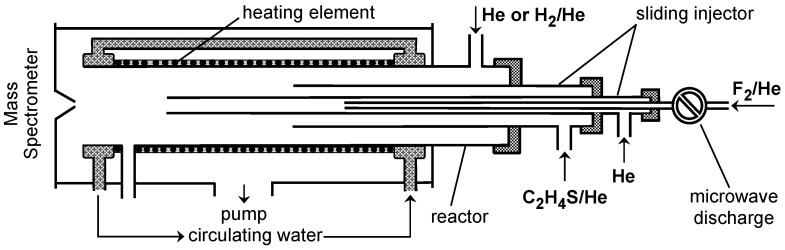
Configuration of the high-temperature flow reactor used in the study of reaction (1).

The reaction of F_2_ with C_2_H_4_S was studied at a total pressure of 2 Torr of He and at temperatures ranging from 220 to 800 K. The configuration of the flow reactors used in the experiments is shown in Figure 1 and Figure S1 (Supplementary Materials). It should be noted that the microwave discharge shown in Figure 1 and Figure S1 was only used in the HF calibration experiments (see experimental section), but was turned off during the kinetic study of the title reaction.

### 2.1. Products of Reaction (1)

#### 2.1.1. Measurements of HF Yield

HF and C_2_H_3_SF were identified as primary products of reaction (1):

F_2_ + C_2_H_4_S → HF + C_2_H_3_SF

Both species were monitored by mass spectrometry on their parent peaks at *m*/*z* = 38 (F_2_^+^) and 78 (C_2_H_3_SF^+^), respectively. Experiments to determine the branching ratio of this reactive channel were carried out with an excess of thiirane over F_2_ and consisted of measuring the consumed concentrations of the reactants and those of the two products formed. With the initial C_2_H_4_S concentrations shown in Table 1 and a reaction time of 0.015 to 0.020 s, the consumed fraction of F_2_ (the initial F_2_ concentration was varied by a factor of approximately 10) was ≥ 90%. The initial concentrations of the two reactants were comparable, allowing the detection of not only the consumption of F_2_, but also that of the excess reagent, C_2_H_4_S.

**Figure 2:**
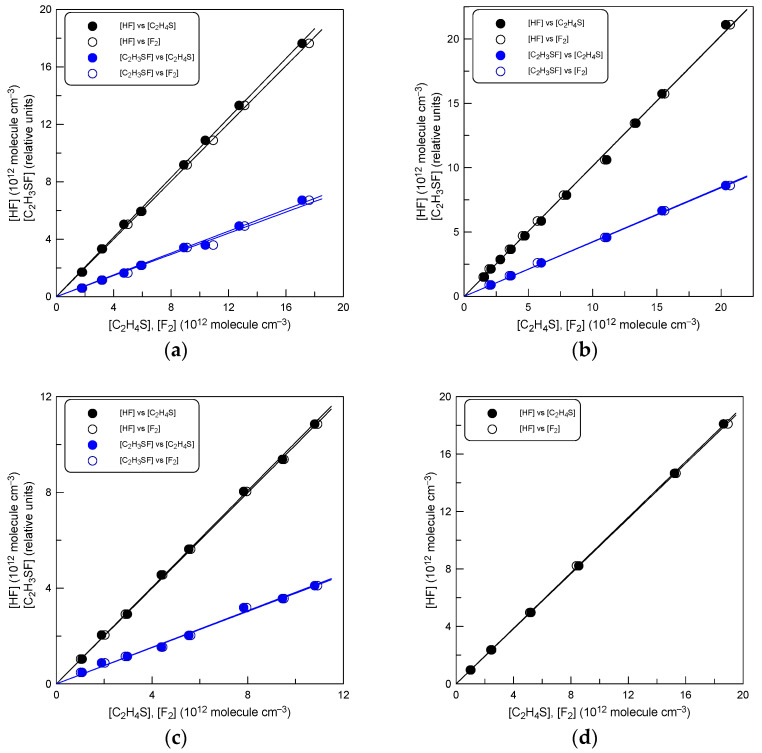
Concentration of the products, HF and C_2_H_3_SF, formed in reaction (1) as a function of the consumed concentration of the reactants, C_2_H_4_S and F_2_: (**a**) T = 220 K; (**b**) T = 298 K; (**c**) T = 500 K; and (**d**) T = 800 K.

Examples of the experimental data observed in these experiments are shown in Figure 2, where the formed concentrations of the reaction products are plotted against the [F_2_] and [C_2_H_4_S] consumed. Note that the concentrations of C_2_H_3_SF in Figure 2 are presented in relative units; absolute concentrations were measured only for HF. The yields of HF determined from the slopes of the black lines in Figure 2 ([HF]/Δ[F_2_] and [HF]/Δ[C_2_H_4_S]) at different temperatures are listed in Table 1. The results show that one molecule of HF is formed per one molecule of F_2_ and C_2_H_4_S consumed. This observation, along with the linearity of the corresponding plots for C_2_H_3_SF, seems to indicate a negligible impact of possible secondary and side reactions under the experimental conditions of the measurements and that the HF + C_2_H_3_SF forming channel is the main, if not only, reaction pathway in the entire temperature range of the study (220–800 K). Combining the statistical uncertainty of measurements with the accuracy of measuring the absolute concentrations of F_2_, C_2_H_4_S and HF of around 5%, a branching ratio equal to unity with an error of 10% can be recommended for the HF forming channel of reaction (1).

As previously noted, all measurements were carried out with an excess of C_2_H_4_S. The fact is that in an excess of F_2_, we observed signs of a secondary reaction of F_2_ with C_2_H_3_SF. The kinetics of C_2_H_3_SF exhibited a characteristic behavior: [C_2_H_3_SF] initially increased to a maximum and then decreased due to the secondary reaction with F_2_. Concurrently, we observed the formation of SF_2_ (at *m*/*z* = 70). Studying this secondary reaction was beyond the scope of this work, so we limited the branching ratio measurements to experiments with excess of C_2_H_4_S, where the secondary chemistry could be neglected. However, it should be noted that at T = 800 K (the highest temperature of the study), we observed evidence of C_2_H_3_SF removal, albeit slowly, even in the absence of F_2_ in the reactor. We are inclined to think that this is due to thermal decomposition of C_2_H_3_SF, although other processes of C_2_H_3_SF removal cannot be ruled out. For this reason, in Figure 2d we do not present the measurements of [C_2_H_3_SF].

The structure of the C_2_H_3_SF formed in reaction (1) was not determined, but some considerations can nevertheless be discussed. Most probably, reaction (1) proceeds through the addition of F_2_ to the sulfur atom followed by the elimination of HF, as proposed by Nelson et al. [6]:

F_2_ + C_2_H_4_S → [C_2_H_4_S(F-F)]* → HF+ C_2_H_3_SF

In this case, the most likely product is CH_2_=CH-SF (ethenyl thiohypofluorite). Mass spectrometry analysis of C_2_H_3_SF (formed in reaction (1)) revealed a prominent fragment peak at *m*/*z* = 51 (SF^+^), which, although indirectly, supports this hypothesis. The presence of this peak in the mass spectrum of other conformers, for example, 2-fluorotiirane or S=CH-CH_2_F, seems to be unlikely. In addition, signals at *m*/*z* = 63 and 64, which can be attributed to C-SF^+^ and CH-SF^+^, respectively, are observed and are consistent with the mass spectrometric fragments of CH_2_=CHSF.

For the analogous reaction of F_2_ with DMS, Turnipseed and Birks [4] observed a reaction product, thought to be H_2_C=S(F)CH_3_, when DMS was in excess over F_2_. This product was found to be destroyed in an excess of F_2_ in the reactor, similar to how CH_2_=CH-SF behaves in the present work. The authors proposed that the reaction proceeds through a charge-transfer complex with subsequent elimination of H and F atoms or of molecular HF. The HF production channel was thought to constitute a small part of the reaction pathway, although there was no experimental evidence for that. In contrast, for the F_2_ reaction with C_2_H_4_S investigated in the present work, a significant contribution of the F atom forming channel can apparently be excluded, given that in an excess of thiirane it was observed that [HF] = Δ[F_2_] = Δ[C_2_H_4_S].

#### 2.1.2. Theoretical Findings

**Figure 3:**
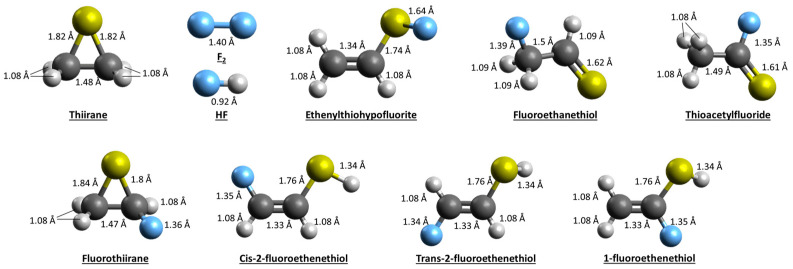
Geometries of the reactants and C_2_H_3_SF isomers/conformers optimized at the MP2/aug-cc-pVTZ level of theory. Sulfur atoms are yellow, fluorine in blue, carbon atoms in dark gray and hydrogen in light gray.

The enthalpies (including Zero-Point Energy, ZPE) and Gibbs free energies of the thiirane reaction with F_2_ were computed for all possible isomers and conformers of the products C_2_H_3_FS (Table 2). These isomers and conformers (for the case of 2-fluoroethenethiol) are shown in Figure 3. The computed reaction enthalpies range from −509.1 kJ mol^−1^ (for the case of thioacetylfluoride) to −409.9 kJ mol^−1^ (in the case of Ethenylhypofluorite). The Gibbs free energies are in the same order of magnitude, indicating a spontaneous reaction.

From the energies of the different products, we can compute the relative Boltzmann population as function of the temperature using the following equation:

$$Boltzmann\;population=e^{\frac{\Delta H}{RT}}/\sum e^{\frac{\Delta H}{RT}}$$

with ∆*H* being the relative enthalpy, *R* the gas constant and *T* the temperature. The Boltzmann population analysis indicates that thioacetylfluoride is the predominant species formed, with 1-fluoroethenethiol being the second most abundant species. These species could be source of CHSF and CSF fragments but not of the SF fragment, which was observed experimentally. It should be noted that calculations provide insights into the thermochemistry, pointing to a highly exergonic reaction, but not into the kinetics, since the energy barrier of the transition states was not calculated. Given the exergonic nature of the reaction, products that have high energy barriers, or include rearrangements, and could proceed through multiple transition states may not be kinetically favored.

### 2.2. Measurements of the Rate Constant of Reaction (1)

In most experiments, the reaction rate constant was determined from the kinetics of C_2_H_4_S consumption ([C_2_H_4_S]_0_ = (1.5 − 5.0) × 10^11^ molecule cm^−3^), monitored in an excess of F_2_ in the reactor (for concentrations of F_2_ see Table 3).

**Figure 4:**
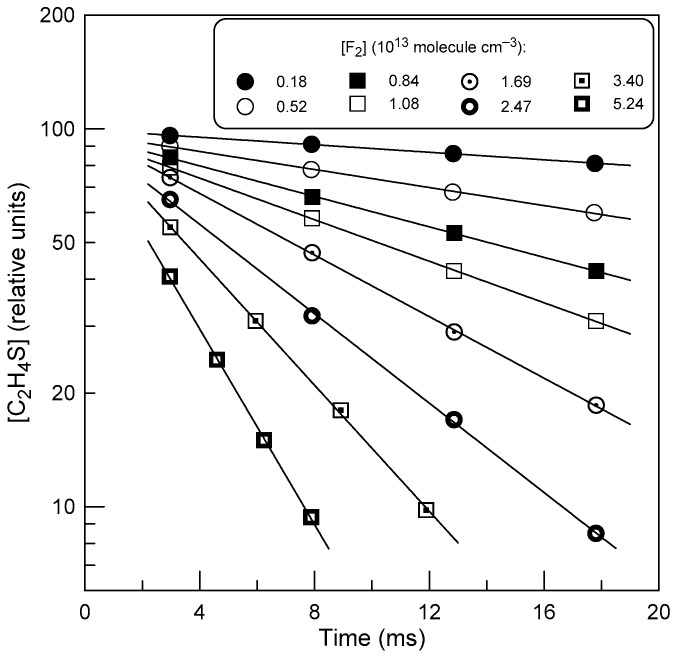
Example of the kinetics of C_2_H_4_S consumption in reaction (1) at different concentrations of F_2_ observed at T = 325 K.

Examples of observed C_2_H_4_S decays are shown in Figure 4. The temporal profiles of C_2_H_4_S were fitted to an exponential function [C_2_H_4_S] = [C_2_H_4_S]_0_ × exp(−*k*_1_′ × t), where [C_2_H_4_S]_0_ and [C_2_H_4_S] are the initial and time-dependent concentrations of thiirane, respectively, and *k*_1_′ = *k*_1_ × [F_2_] is the pseudo-first-order rate constant. The diffusion corrections made in [7] to the *k*_1_′ values measured in this way were less than 10%.

**Figure 5:**
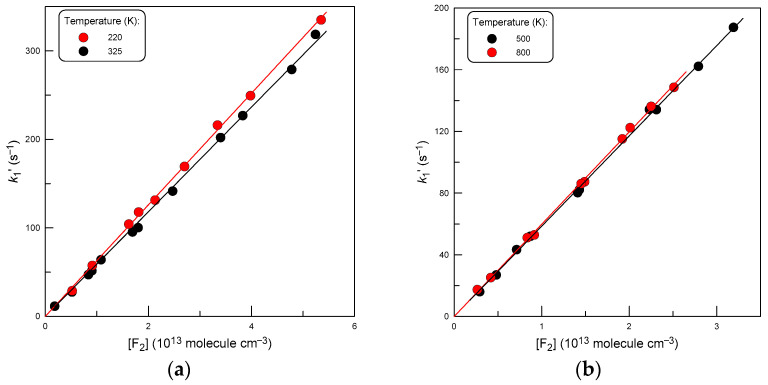
Pseudo-first-order rate constant, *k*_1′_ = *k*_1_ × [F_2_], as a function of F_2_ concentration at different temperatures: (**a**) T = 220 and 325 K; (**b**) T = 500 and 800 K.

Examples of second-order plots measured at different temperatures are shown in Figure 5. A linear least-square fit through the origin of the *k*_1_′ data as a function of [F_2_] provides the rate constant of reaction (1) at the corresponding temperature. All the results obtained for *k*_1_ are given in Table 3.

**Figure 6:**
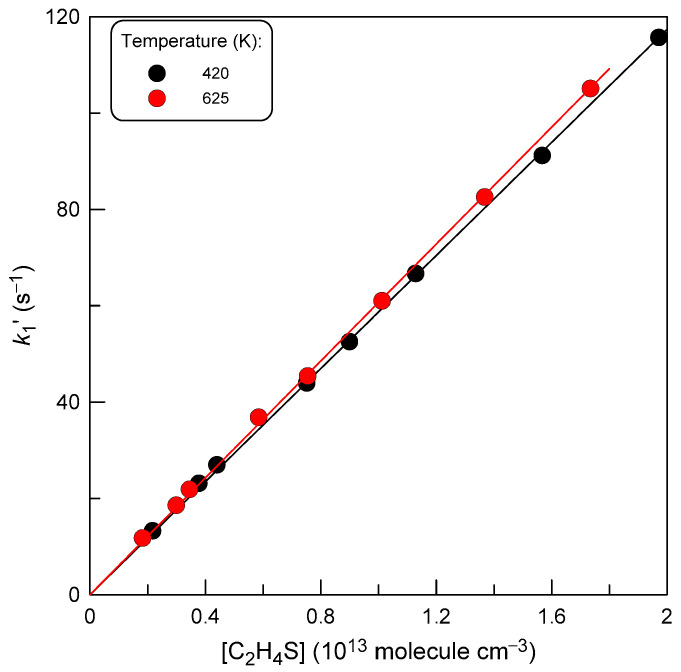
Pseudo-first-order rate constant, *k*_1′_ = *k*_1_ × [C_2_H_4_S], as a function of C_2_H_4_S concentration observed at T = 420 and 625 K.

In some experiments, the rate constant of reaction (1) was determined from the kinetics of F_2_ consumption monitored in an excess of thiirane in the reactor. The initial concentration of F_2_ in these experiments was ≤ 10^12^ molecule cm^−3^. The observed C_2_H_4_S consumption (within a few %) was taken into account by using the average C_2_H_4_S concentration over the reaction zone. Examples of second-order plots and final values of *k*_1_ obtained in this series of experiments are shown in Figure 6 and Table 3, respectively.

**Figure 7:**
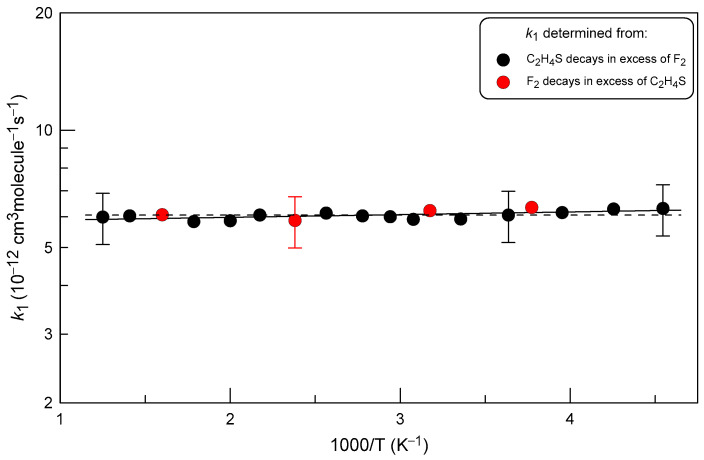
Temperature dependence of the rate constant of reaction (1). Partially shown error bars correspond to estimated total uncertainty of 15% on the measurements of *k*_1_.

The results of all *k*_1_ measurements are summarized in Figure 7.

It can be noted that there is an excellent agreement between the data obtained under different experimental conditions, from F_2_ and C_2_H_4_S kinetics in excess of C_2_H_4_S and F_2_, respectively. Fitting the dependence of *k*_1_ on temperature to the exponential function (solid line in Figure 6) gives the following Arrhenius expression:

*k*_1_ = (5.79 ± 0.17)×10^−2^ exp(−(16 ± 10)/T) cm^3^ molecule^−1^ s^−1^

at T = 220–800 K with 2σ uncertainties representing the precision of the fit. We estimate this expression to be accurate within an overall 2σ uncertainty of 15% over the investigated temperature range. Considering the virtual independence of the rate constant of temperature, the temperature independent value of

*k*_1_ = (6.05 ± 0.90) × 10^−12^ cm^3^ molecule^−1^ s^−1^

can be recommended for the rate constant of reaction (1) (dashed line in Figure 6) in the temperature range (220−800) K. The observed temperature independence of *k*_1_ appears to be consistent with a reaction mechanism consisting of barrier-free formation of an intermediate followed by its decomposition into reactants or reaction products.

Turnipseed and Birks [4] in their study of the F_2_+DMS reaction speculated that the reaction can be initiated by the transfer of an electron from the sulfur compound to F_2_, forming a charge-transfer complex. Considering that the electron transfer process must be either exothermic or thermoneutral for the reaction to proceed at a measurable rate they calculated a critical distance, *r*_c_ = 14.4/(IP(reactant) − EA(F_2_)), which corresponds to the maximum distance at which the charge-transfer complex can be stable and the electron can be transferred (EA(F_2_): electron affinity of molecular fluorine; IP(reactant): ionization potential of the second reactant). By analyzing the *r*_c_ for F_2_ interactions with a number of compounds, the authors estimated that a critical distance greater than 2.3 Å is required for the reaction to occur [4]. The present data for the reaction of F_2_ with thiirane are consistent with this reasoning, given that *r*_c_ = 2.4 Å (calculated with IP(C_2_H_4_S) = 9.05 eV [8] and EA(F_2_) = 3.08 eV [9]) and a relatively high value was measured for the reaction rate constant.

## 3. Materials and Methods

### 3.1. Experimental

The experimental setup consisted of a discharge flow reactor combined with a modulated molecular beam mass spectrometer with electron impact ionization operated at 30 eV energy (Figure 1) [10,11]. The reaction time was determined by the position of the movable injector relative to the sampling cone of the mass spectrometer; changing its position makes it possible to vary the reaction time. Linear flow velocities in the reactor ranged from 1730 to 2400 cm s^−1^. The chemical composition of the reactive system was monitored by sampling gas-phase molecules from the flow reactor and detecting them with a mass spectrometer. All species involved were detected at their parent peaks.

Two flow reactors were used in this study to cover a wide temperature range for kinetic measurements. The first reactor, operated at high temperatures (315–800 K), consisted of an electrically heated quartz tube (45 cm length and 2.5 cm i.d.) with water-cooled attachments (Figure 1) [12]. The temperature in the reactor was measured with a K-type thermocouple positioned in the middle of the reactor in contact with its outer surface [12]. The second flow reactor (Figure S1) used at lower temperatures (220–325 K) consisted of a Pyrex tube (45 cm length and 2.4 cm i.d.); temperature regulation was achieved by circulating thermostated ethanol. The walls of the Pyrex reactor, as well as the mobile injector of fluorine atoms, were coated with halocarbon wax to prevent the reaction of the F atom with the glass surface.

The absolute concentrations of F_2_, H_2_ and C_2_H_4_S were calculated from their flow rates, obtained from pressure drop measurements of their mixtures in He stored in calibrated volume flasks. Absolute calibration of the mass spectrometer to HF was carried out by titrating a known concentration of H_2_ with an excess of F atoms ([HF] = [H_2_]) in a fast reaction:

F + H_2_ → HF + H (2)

*k*_2_ = 1.24 × 10^−10^ exp(−507/T) cm^3^ molecule^−1^ s^−1^ (T = 220–960 K) [13]. Fluorine atoms in these experiments were generated in a microwave discharge of trace amounts of F_2_ in He. It was verified by mass spectrometry that more than 95% of F_2_ was dissociated in the microwave discharge. To reduce F atom reactions with the glass surface inside the microwave cavity, a ceramic (Al_2_O_3_) tube was inserted in this part of the injector.

The purities of the gases used were as follows: He (>99.9995%, Alphagaz, Air Liquide, Paris, France), passed through liquid nitrogen trap; H_2_ (> 99.998%, Alphagaz); and F_2_, 5% in helium (Alphagaz); C_2_H_4_S (Merck, Merck SA, Lyon, France), 98%.

### 3.2. Computational Methodology

In order to select the correct methodology to perform the computation, a thorough benchmark was carried out. Based on Vila et al. [14], we used density functional theory (DFT) with a B3LYP function in comparison with the Schrödinger-based MP2 method and CCSD method, which is used as reference, having the highest level of accuracy among the benchmarked methods. In practice, DFT, MP2 and CCSD calculations were performed with the Gaussian 16 software [15], while the more time-consuming CCSD(T)-CBS calculations were carried out using the Molpro 2023.2.0 software [16,17,18].

B3LYP and MP2 methods were compared using the same basis set (6-311++G(3d2f,3p2d)) as the CCSD methodology. The criteria to determine the accuracy of B3LYP and MP2 compared to CCSD were based on the geometries of the molecules and their energies once corrected with single-point computation at the same level of methodology as when using CCSD(T) with a CBS correction (aug-cc-pVTZ:aug-cc-pVQZ). In Figure S2, the energy differences are introduced and it is shown that only the MP2 method remains with differences under chemical accuracy (4.18 kJ/mol). Figures S3–S10 illustrate the geometry differences for each product, showing negligible discrepancies in bond lengths (maximum of 0.02 Å), indicating the minimal impact of the method on this parameter. Concerning angles, small differences are observed with a maximum of 1.28 degrees. However, we can start to observe some indication that MP2 is slightly better than B3LYP to reproduce the angles. Finally, concerning dihedral angles, strong differences can be observed in the case of the B3LYP methodology compared to MP2. Indeed, in the case of cis-2-fluoroethenethiol, ethenylthiohypofluorite and fluoroethanethial some dihedral angles increase a lot (up to 127.55 degrees) in the case of B3LYP. Such differences indicate a clear change in the configuration of the molecule. In MP2, the differences are up to 7.02 degrees, which is more reasonable. In conclusion, the MP2 methodology seems more reliable in comparison to the CCSD method. The computational cost increases slightly compared to DFT but is still reasonable compared to the CCSD methodology, which is prohibitively expensive.

Using both B3LYP and MP2, which are reasonable in terms of computational cost, the 6-311++G(3d2f,3p2d) and aug-cc-pVDZ were compared to the aug-cc-pVTZ basis set, which is the largest among the three. Geometries and energies are compared, as they are in the benchmark of the method. In Figure S11, energy differences clearly indicate that basis set size has an effect, as the energy is above chemical accuracy in the case of 6-311++G(3d2f,3p2d) and for ethenylthiohypofluorite with the aug-cc-pVDZ. Figures S12–S19 introduce geometry differences. It is evident that the 6-311++G(3d2f,3p2d) basis set struggles to accurately represent bonds involving sulfur and fluorine atoms. It is even more remarkable in the case of ethenylthiohypofluorite, where the error is around 0.16 Å (B3LYP) and 0.18 Å (MP2). These differences can significantly impact the calculated energies. Dihedral angles are notably impacted by the size of the basis set for both methods. Therefore, the larger aug-cc-pVTZ basis set is recommended.

## Figures and Tables

**Table 1:** Experimental conditions and results of the measurements of HF yield in reaction (1).

*T* (K)	[C_2_H_4_S]_0_ *^a^*	Δ[F_2_] *^b^*	Δ[HF]/Δ[C_2_H_4_S] *^c^*	Δ[HF]/Δ[F_2_] *^d^*
220	3.5–3.8	0.18–1.80	1.04 ± 0.01	1.01±0.01
253	4.5–5.0	0.12–2.08	1.02 ± 0.01	1.01±0.01
298	3.2–5.0	0.19–2.07	1.01 ± 0.02	1.01±0.01
325	4.5–5.0	0.15–2.04	1.06 ± 0.01	1.05 ± 0.01
360	2.0–3.8	0.09–1.51	0.98 ± 0.01	0.95 ± 0.01
500	3.1–3.5	0.10–1.09	1.01 ± 0.01	1.00 ± 0.01
670	2.0–2.5	0.08–0.72	1.02 ± 0.01	1.00 ± 0.01
800	3.32–4.0	0.10–1.89	0.97 ± 0.01	0.96 ± 0.01

*^a^* Initial concentration of C_2_H_4_S (units of 10^13^ molecule cm^−3^); *^b^* consumed concentration of F_2_ (units of 10^13^ cm^3^ molecule^−1^ s^−1^); *^c^* ratio of [HF] formed to [C_2_H_4_S] consumed; *^d^* ratio of [HF] formed to [F_2_] consumed. For HF yield statistical 2σ uncertainty is given, total estimated uncertainty is 10%.

**Table 2:** Summary of the enthalpies (in kJ/mol, including the ZPE correction), Gibbs free energies and Boltzmann population for the isomers and conformers at temperature of 298, 500 and 1000 K, computed at the CCSD(T)-CBS/(aug-cc-pVTZ:aug-cc-pVQZ)//MP2/aug-cc-pVTZ level.

Molecule	∆G (kJ/mol)	∆H (kJ/mol)	Boltzmann Population T = 298 K	Boltzmann Population T = 500 K	Boltzmann Population T = 1000 K
1-fluoroethenethiol	−470.7	−473.1	2.0 × 10^−7^	1.0 × 10^−4^	9.8 × 10^−3^
Cis-2-fluoroethenethiol	−467.1	−467.8	2.4 × 10^−8^	2.9 × 10^−5^	5.2 × 10^−3^
Ethenylthyohypofluorite	−409.9	−411.3	3.0 × 10^−18^	3.6 × 10^−11^	5.9 × 10^−6^
Fluoroethanethiol	−456.3	−456.6	2.6 × 10^−10^	1.9 × 10^−6^	1.4 × 10^−3^
Fluorothiirane	−462.0	−466.2	1.3 × 10^−8^	2.0 × 10^−5^	4.3 × 10^−3^
Thioacetylfluoride	−509.1	−511.3	1	1	9.8 × 10^−1^
Trans-2-fluoroethenethiol	−463.4	−465.5	9.3 × 10^−9^	1.6 × 10^−5^	4.0 × 10^−3^

**Table 3:** Summary of the measurements of the rate constant of reaction (1).

*T* (K) *^a^*	Excess Reactant	[Excess Reactant] *^b^*	*k*_1_(±2σ) *^c^*	Reactor Surface *^d^*
220	F_2_	0.52–5.35	6.30 ± 0.08	HW
235	F_2_	0.39–4.70	6.28 ± 0.09	HW
253	F_2_	0.39–5.53	6.15 ± 0.06	HW
265	C_2_H_4_S	0.24–4.82	6.34 ± 0.05	HW
275	F_2_	0.30–4.60	6.06 ± 0.06	HW
298	F_2_	0.36–5.45	5.92 ± 0.07	HW
315	C_2_H_4_S	0.23–2.31	6.22 ± 0.09	Q
325	F_2_	0.18–5.24	5.91 ± 0.09	HW
340	F_2_	0.20–4.00	6.00 ± 0.06	Q
360	F_2_	0.36–4.02	6.03 ± 0.07	Q
390	F_2_	0.35–4.68	6.13 ± 0.10	Q
420	C_2_H_4_S	0.22–1.94	5.87 ± 0.05	Q
460	F_2_	0.36–3.69	6.06 ± 0.12	Q
500	F_2_	0.29–3.19	5.86 ± 0.06	Q
560	F_2_	0.22–3.30	5.83 ± 0.11	Q
625	C_2_H_4_S	0.18–1.73	6.07 ± 0.06	Q
710	F_2_	0.21–2.88	6.03 ± 0.09	Q
800	F_2_	0.27–2.51	5.99 ± 0.06	Q

*^a^* 8–12 decay traces at each temperature; *^b^* units of 10^13^ molecule cm^−3^; *^c^* units of 10^−12^ cm^3^ molecule^−1^ s^−1^, statistical 2σ uncertainty is given, total estimated uncertainty of *k*_1_ is 15%; *^d^* HW: halocarbon wax; Q: quartz.

## Data Availability

The data supporting reported results are available in this article.

## Supplementary Materials

The following supporting information can be downloaded at: https://www.mdpi.com/article/10.3390/molecules29153636/s1, Figure S1: configuration of the low-temperature flow reactor; Figure S2: single-point energy differences between B3LYP, MP2 and CCSD methods; Figures S3–S10: bond length differences calculated with different methods; Figure S11: single-point energy differences for different basis set sizes; Figures S12–S19: geometry differences between different basis sets; Figure S20: Boltzmann relative distribution of the reaction products as a function of temperature; optimized geometry; inputs.
