# Supplementary material for: Experimental and Theoretical Study of the Reaction of F2 with Thiirane
Source: Molecules. 2024 Jul 31;29(15):3636. doi: 10.3390/molecules29153636 (PMC11314507; doi:10.3390/molecules29153636)
Supplement: Supplementary file 1 [file molecules-29-03636-s001.zip › molecules-3117048-supplementary.pdf]

Supporting Information

Experimental and theoretical study of the reaction of F_2 with thiirane

Yuri Bedjanian [1,*], Antoine Roose [2], Valérie Vallet [3], Manolis N. Romanias [2]

[1] Institut de Combustion, Aérothermique, Réactivité et Environnement (ICARE), CNRS 45071 Orléans, France; yuri.bedjanian@cnrs-orleans.fr
[2] IMT Nord Europe, Institut Mines-Télécom, Univ. Lille, Centre for Energy and Environment, F-59000 Lille, France; antoine.roose@imt-nord-europe.fr, emmanouil.romanias@imt-nord-europe.fr
[3] Univ. Lille, CNRS, UMR 8523-PhLAM-Physique des Lasers Atomes et Molécules, F-59000 Lille, France; valerie.vallet@univ-lille.fr
* Correspondence: yuri.bedjanian@cnrs-orleans.fr

Number of pages (including this one): 29

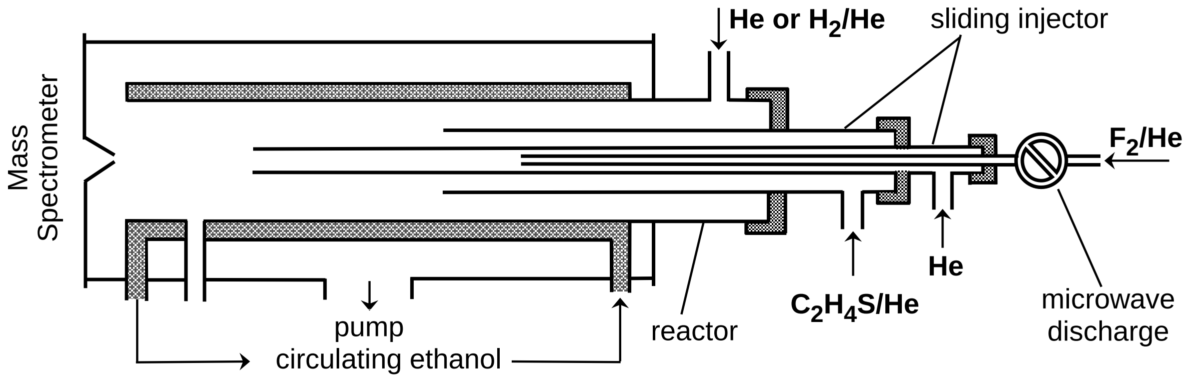

Figure S1. Configuration of the low temperature flow reactor used in the study of reaction (1).

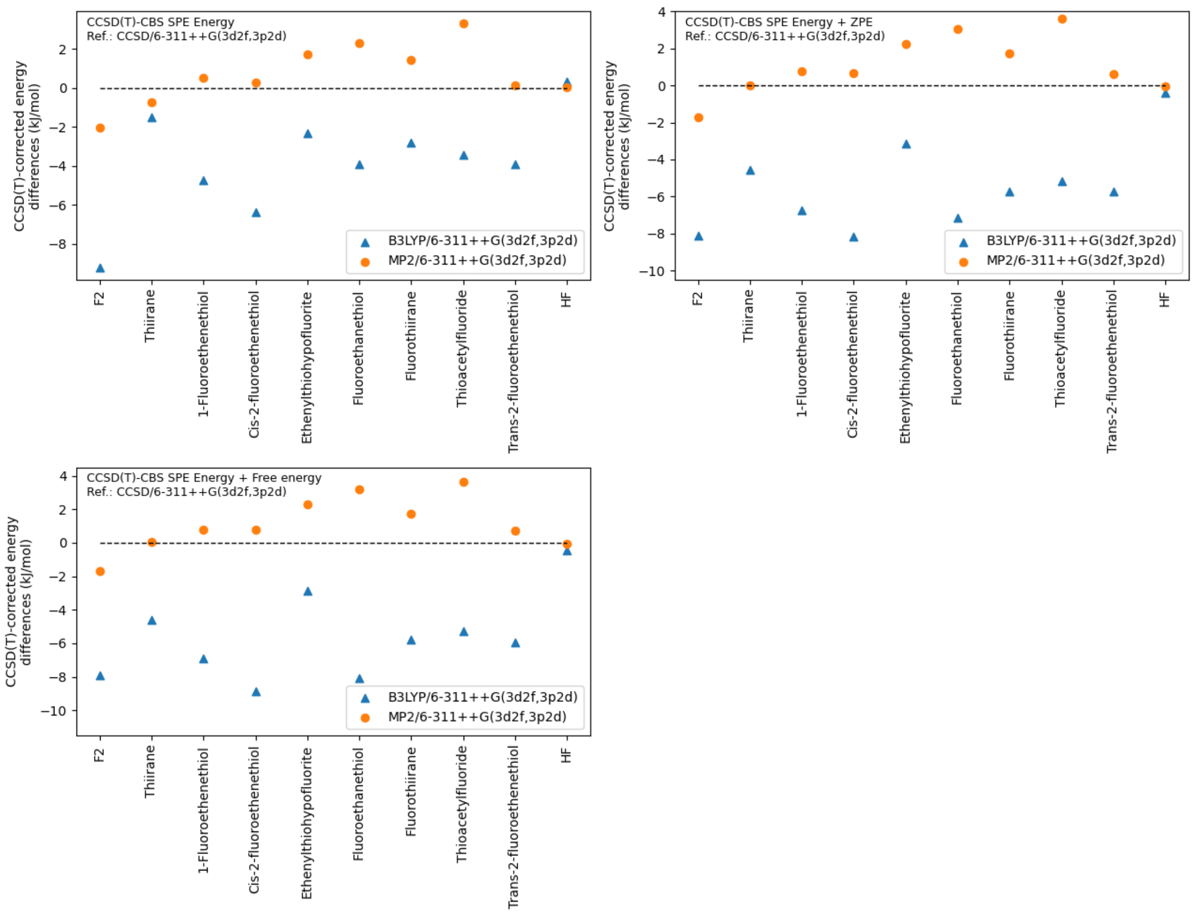

Figure S2. Top left: CCSD(T)-CBS/(aug-cc-pVTZ:aug-cc-pVQZ) single point energy (SPE) differences between B3LYP, MP2 and CCSD (reference) for every molecule. Top right: CCSD(T)-CBS/(aug-cc-pVTZ:aug-cc-pVQZ) single point energy (SPE) + Zero Point Energy (ZPE) differences between B3LYP, MP2 and CCSD (reference) for every molecule. Bottom left: CCSD(T)-CBS/(aug-cc-pVTZ:aug-cc-pVQZ) single point energy (SPE) + thermal free energy differences between B3LYP, MP2 and CCSD (reference) for every molecule. The geometry optimization has been performed at (B3LYP, MP2 or CCSD)/6-311++G(3d2f,3p2d) level in all cases.

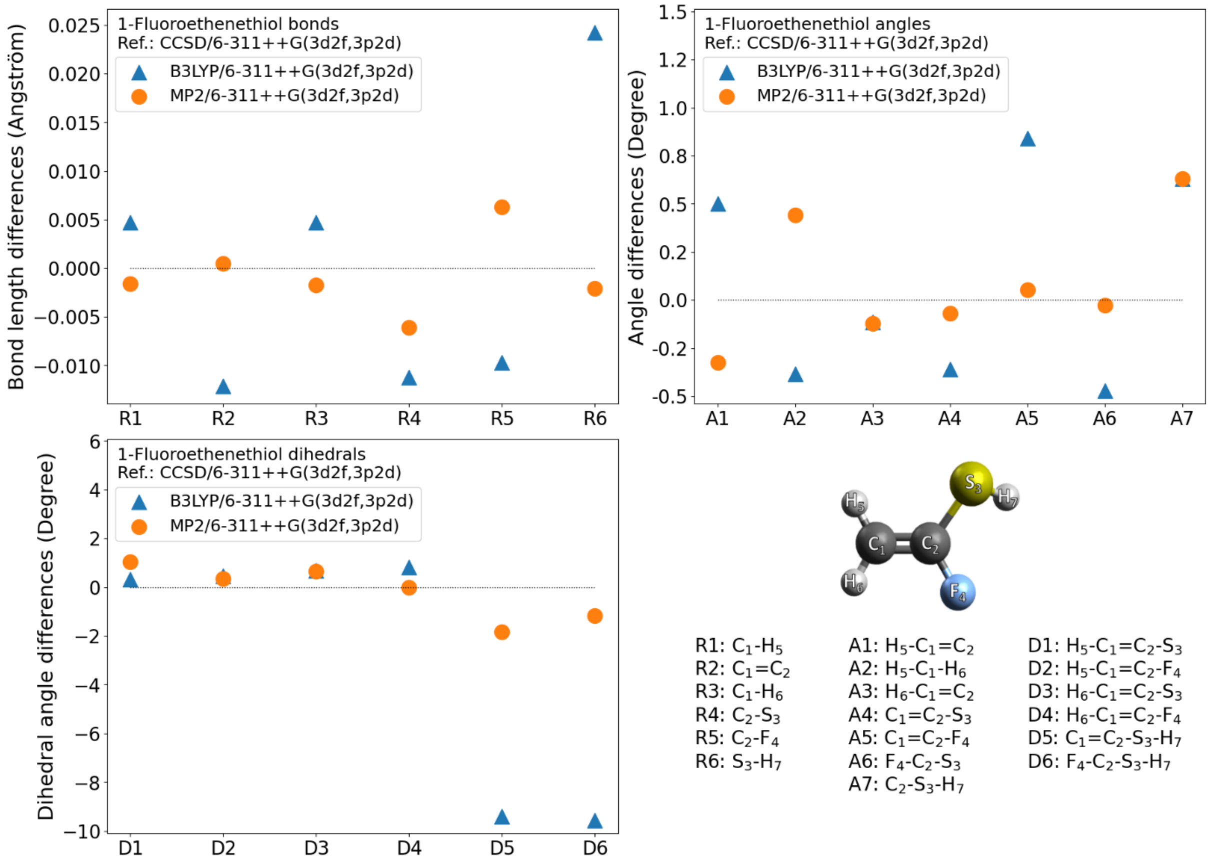

Figure S3. Top left: bond length differences between MP2/6-311++G(3d2f,3p2d) (Blue) and B3LYP/6-311++G(3d2f,3p2d) (Orange) with CCSD /6-311++G(3d2f,3p2d) level of theory in the case of 1-Fluoroethenethiol. Top right: angle differences between MP2/6-311++G(3d2f,3p2d) (Blue) and B3LYP/6-311++G(3d2f,3p2d) (Orange) with CCSD /6-311++G(3d2f,3p2d) level of theory in the case of 1-Fluoroethenethiol. Bottom left: dihedral angle differences between MP2/6-311++G(3d2f,3p2d) (Blue) and B3LYP/6-311++G(3d2f,3p2d) (Orange) with CCSD /6-311++G(3d2f,3p2d) level of theory in the case of 1-Fluoroethenethiol.

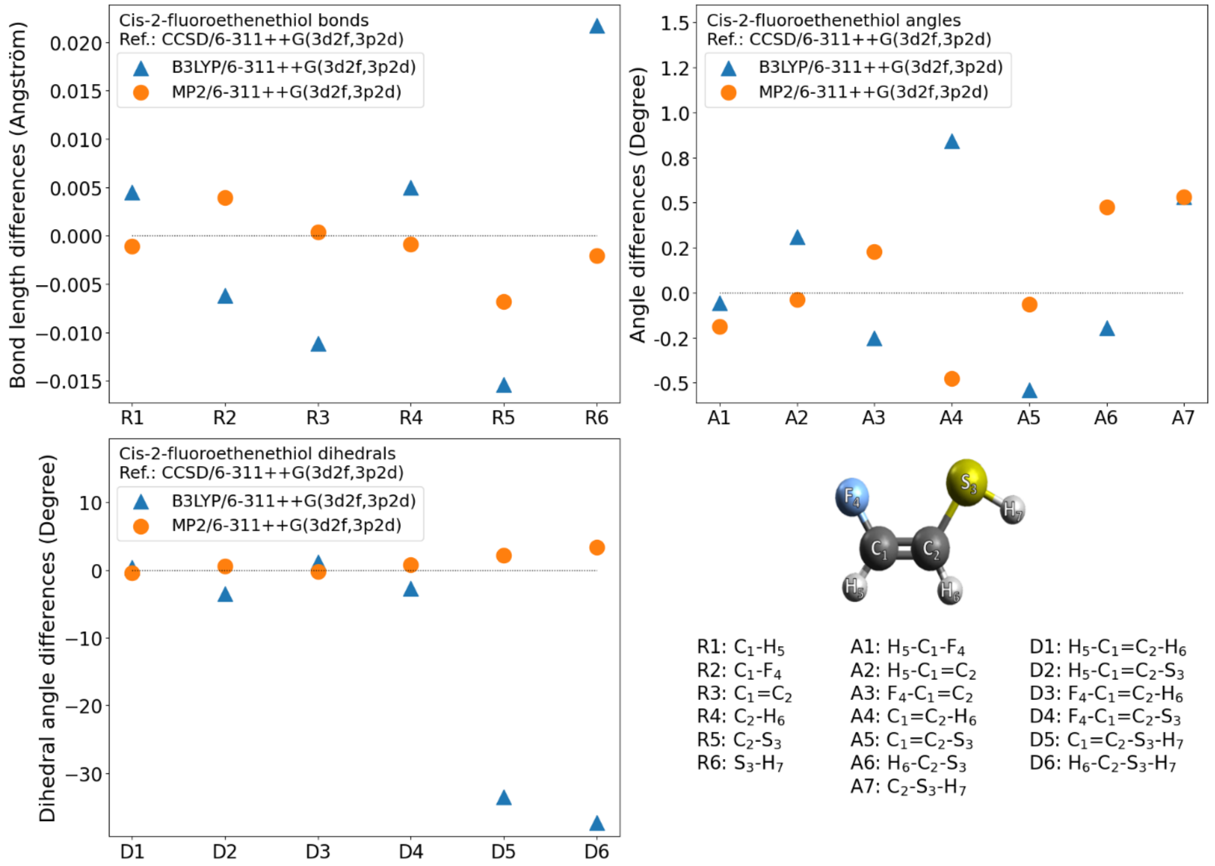

Figure S4. Top left: bond length differences between MP2/6-311++G(3d2f,3p2d) (Blue) and B3LYP/6-311++G(3d2f,3p2d) (Orange) with CCSD /6-311++G(3d2f,3p2d) level of theory in the case of Cis-2-fluoroethenethiol. Top right: angle differences between MP2/6-311++G(3d2f,3p2d) (Blue) and B3LYP/6-311++G(3d2f,3p2d) (Orange) with CCSD /6-311++G(3d2f,3p2d) level of theory in the case of Cis-2-fluoroethenethiol. Bottom left: dihedral angle differences between MP2/6-311++G(3d2f,3p2d) (Blue) and B3LYP/6-311++G(3d2f,3p2d) (Orange) with CCSD /6-311++G(3d2f,3p2d) level of theory in the case of Cis-2-fluoroethenethiol.

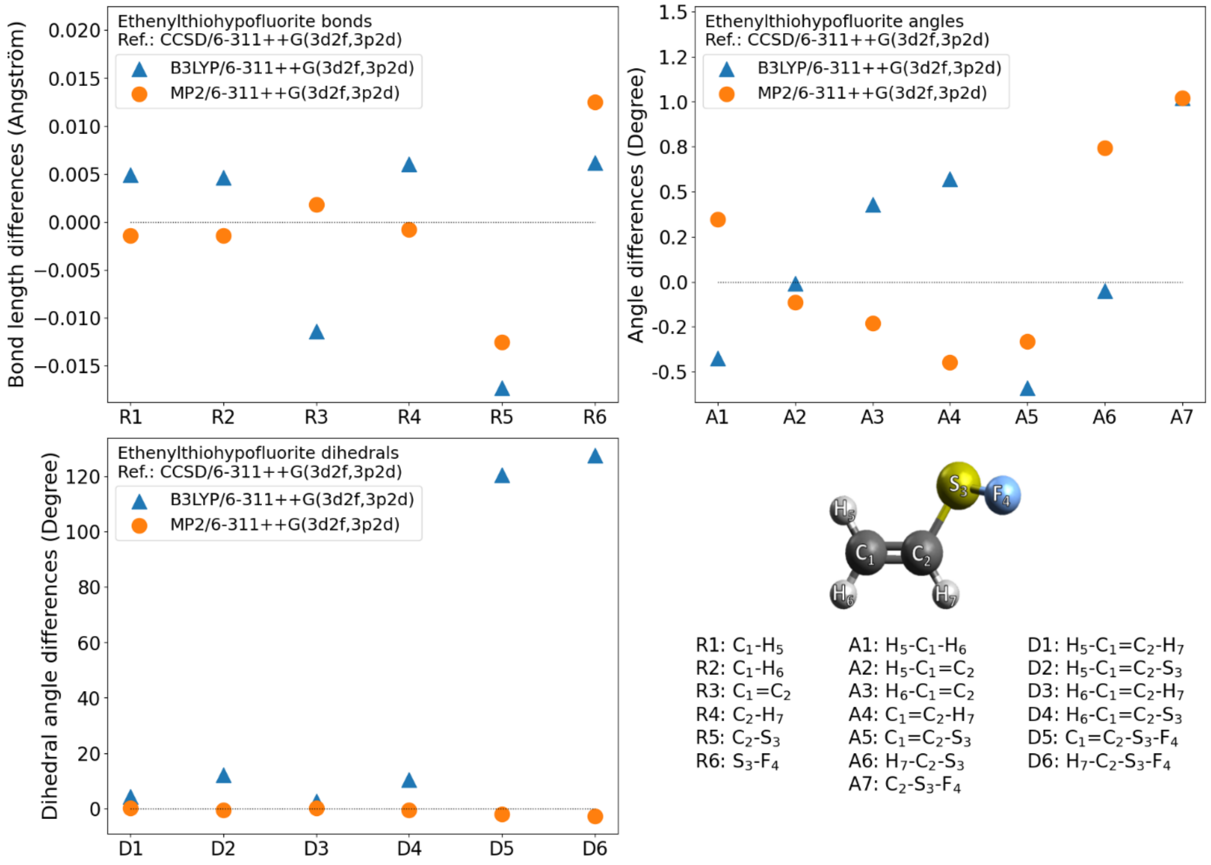

Figure S5. Top left: bond length differences between MP2/6-311++G(3d2f,3p2d) (Blue) and B3LYP/6-311++G(3d2f,3p2d) (Orange) with CCSD /6-311++G(3d2f,3p2d) level of theory in the case of Ethenylthiohypofluorite. Top right: angle differences between MP2/6-311++G(3d2f,3p2d) (Blue) and B3LYP/6-311++G(3d2f,3p2d) (Orange) with CCSD /6-311++G(3d2f,3p2d) level of theory in the case of Ethenylthiohypofluorite. Bottom left: dihedral angle differences between MP2/6-311++G(3d2f,3p2d) (Blue) and B3LYP/6-311++G(3d2f,3p2d) (Orange) with CCSD /6-311++G(3d2f,3p2d) level of theory in the case of Ethenylthiohypofluorite.

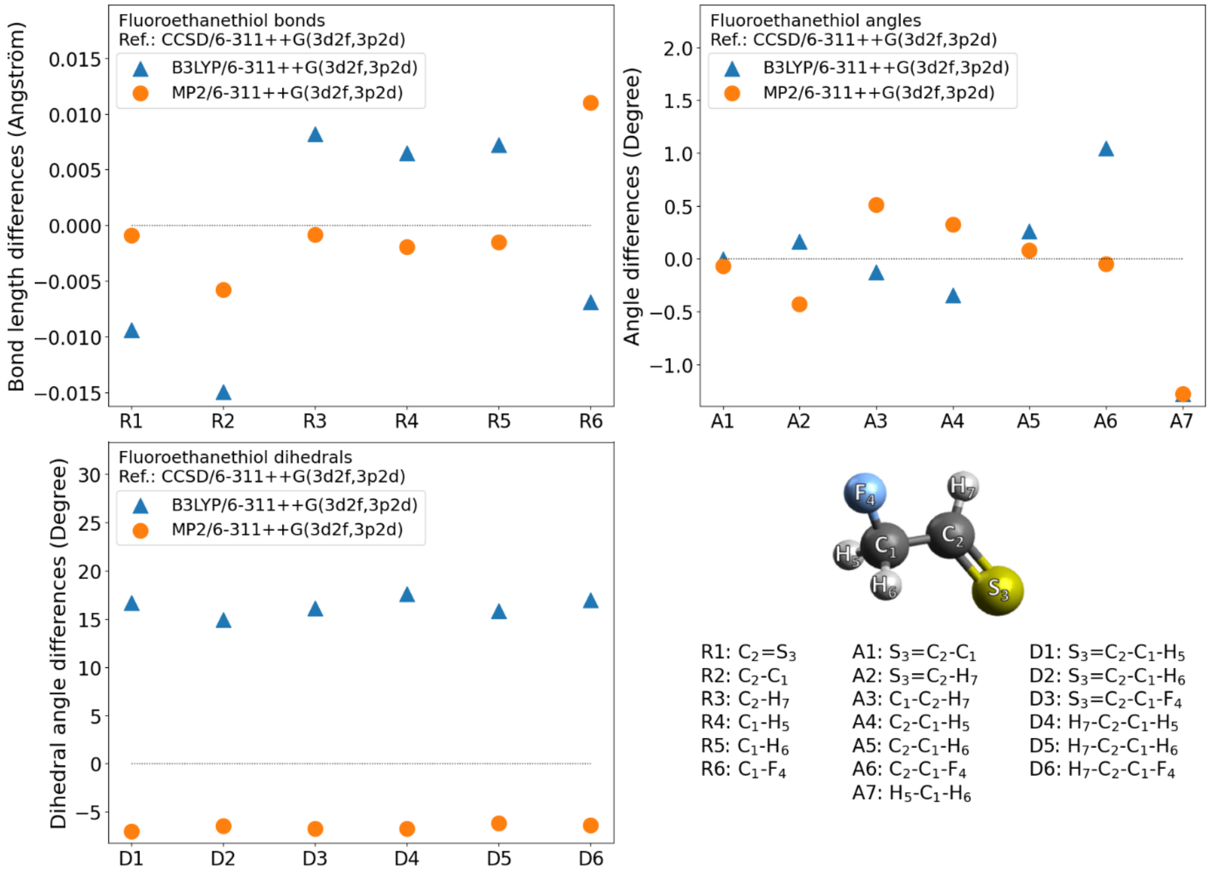

Figure S6. Top left: bond length differences between MP2/6-311++G(3d2f,3p2d) (Blue) and B3LYP/6-311++G(3d2f,3p2d) (Orange) with CCSD /6-311++G(3d2f,3p2d) level of theory in the case of Fluoroethanethiol. Top right: angle differences between MP2/6-311++G(3d2f,3p2d) (Blue) and B3LYP/6-311++G(3d2f,3p2d) (Orange) with CCSD /6-311++G(3d2f,3p2d) level of theory in the case of Fluoroethanethiol. Bottom left: dihedral angle differences between MP2/6-311++G(3d2f,3p2d) (Blue) and B3LYP/6-311++G(3d2f,3p2d) (Orange) with CCSD /6-311++G(3d2f,3p2d) level of theory in the case of Fluoroethanethiol.

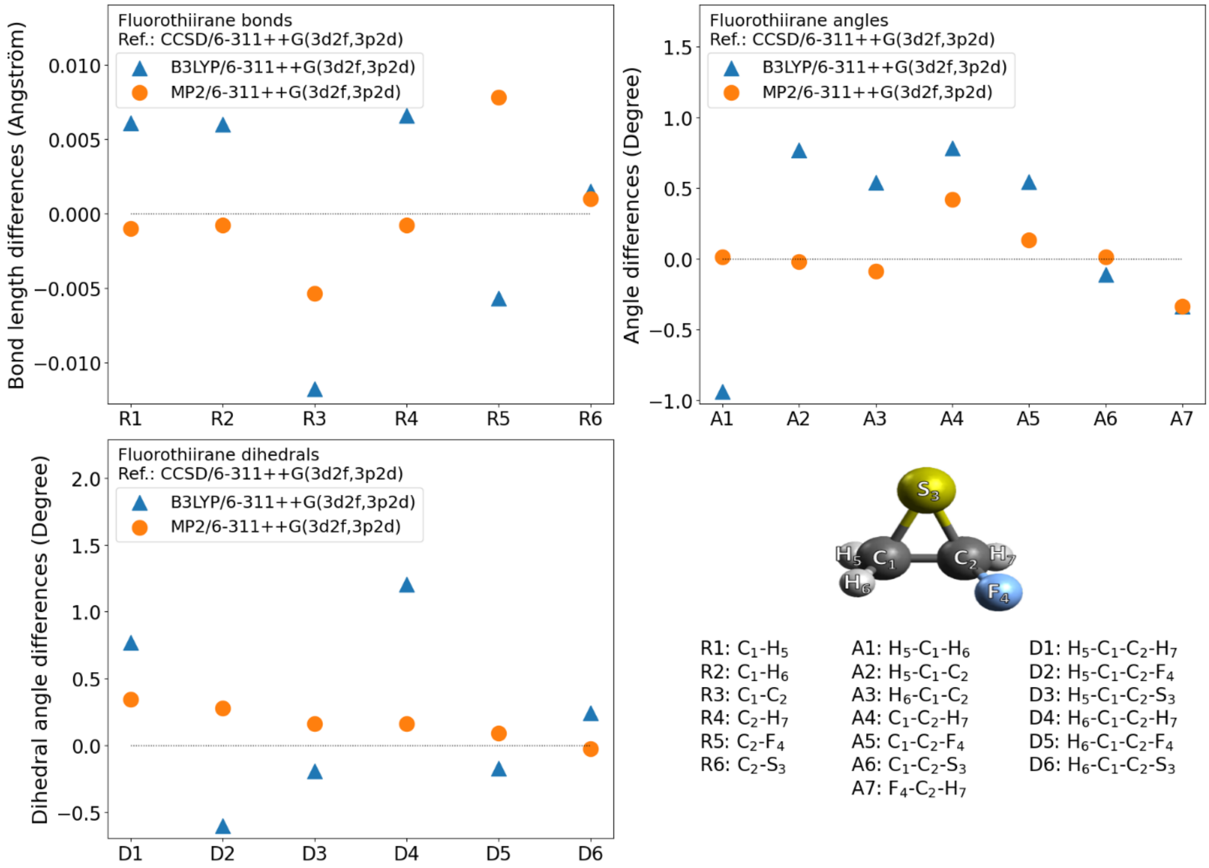

Figure S7. Top left: bond length differences between MP2/6-311++G(3d2f,3p2d) (Blue) and B3LYP/6-311++G(3d2f,3p2d) (Orange) with CCSD /6-311++G(3d2f,3p2d) level of theory in the case of Fluorothiirane. Top right: angle differences between MP2/6-311++G(3d2f,3p2d) (Blue) and B3LYP/6-311++G(3d2f,3p2d) (Orange) with CCSD /6-311++G(3d2f,3p2d) level of theory in the case of Fluorothiirane. Bottom left: dihedral angle differences between MP2/6-311++G(3d2f,3p2d) (Blue) and B3LYP/6-311++G(3d2f,3p2d) (Orange) with CCSD /6-311++G(3d2f,3p2d) level of theory in the case of Fluorothiirane.

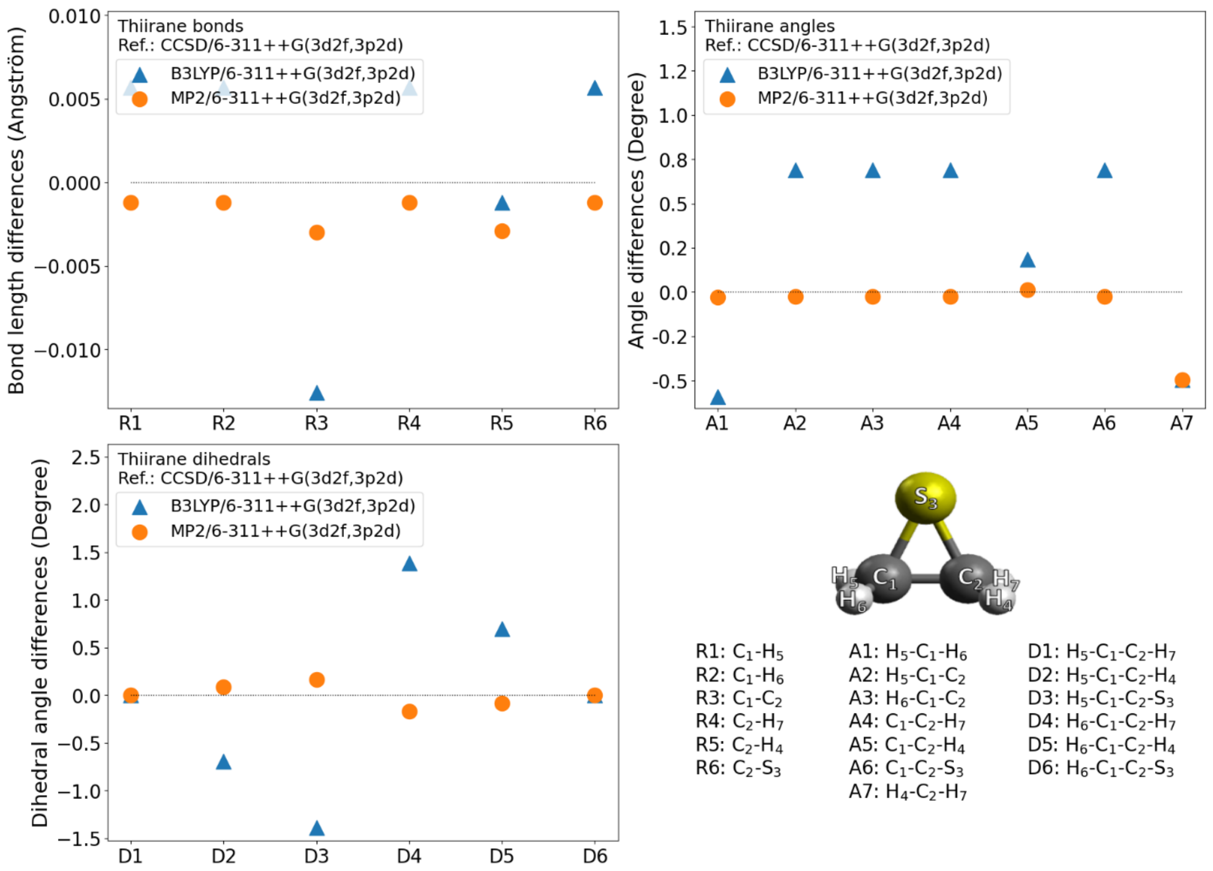

Figure S8. Top left: bond length differences between MP2/6-311++G(3d2f,3p2d) (Blue) and B3LYP/6-311++G(3d2f,3p2d) (Orange) with CCSD /6-311++G(3d2f,3p2d) level of theory in the case of Thiirane. Top right: angle differences between MP2/6-311++G(3d2f,3p2d) (Blue) and B3LYP/6-311++G(3d2f,3p2d) (Orange) with CCSD /6-311++G(3d2f,3p2d) level of theory in the case of Thiirane. Bottom left: dihedral angle differences between MP2/6-311++G(3d2f,3p2d) (Blue) and B3LYP/6-311++G(3d2f,3p2d) (Orange) with CCSD /6-311++G(3d2f,3p2d) level of theory in the case of Thiirane.

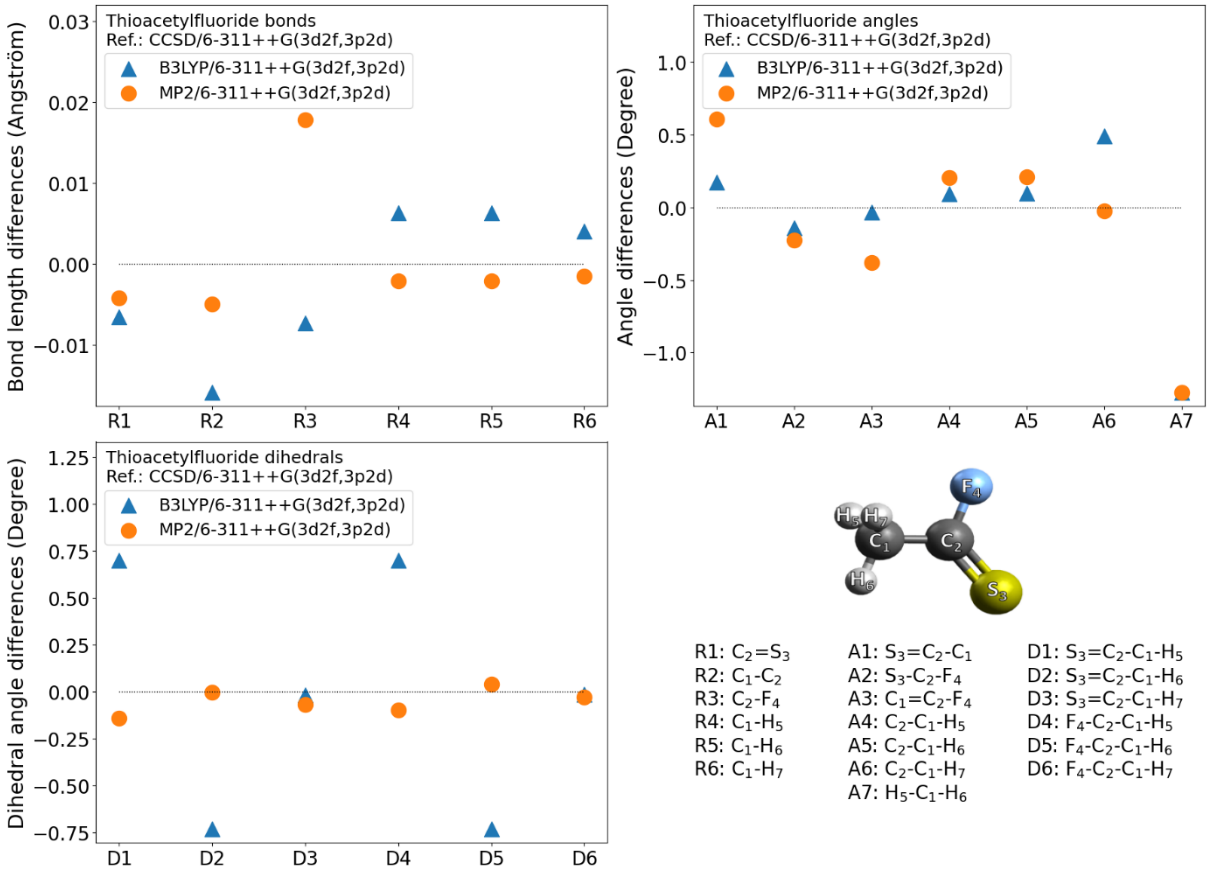

Figure S9. Top left: bond length differences between MP2/6-311++G(3d2f,3p2d) (Blue) and B3LYP/6-311++G(3d2f,3p2d) (Orange) with CCSD /6-311++G(3d2f,3p2d) level of theory in the case of Thioacetylfluoride. Top right: angle differences between MP2/6-311++G(3d2f,3p2d) (Blue) and B3LYP/6-311++G(3d2f,3p2d) (Orange) with CCSD /6-311++G(3d2f,3p2d) level of theory in the case of Thioacetylfluoride. Bottom left: dihedral angle differences between MP2/6-311++G(3d2f,3p2d) (Blue) and B3LYP/6-311++G(3d2f,3p2d) (Orange) with CCSD /6-311++G(3d2f,3p2d) level of theory in the case of Thioacetylfluoride.

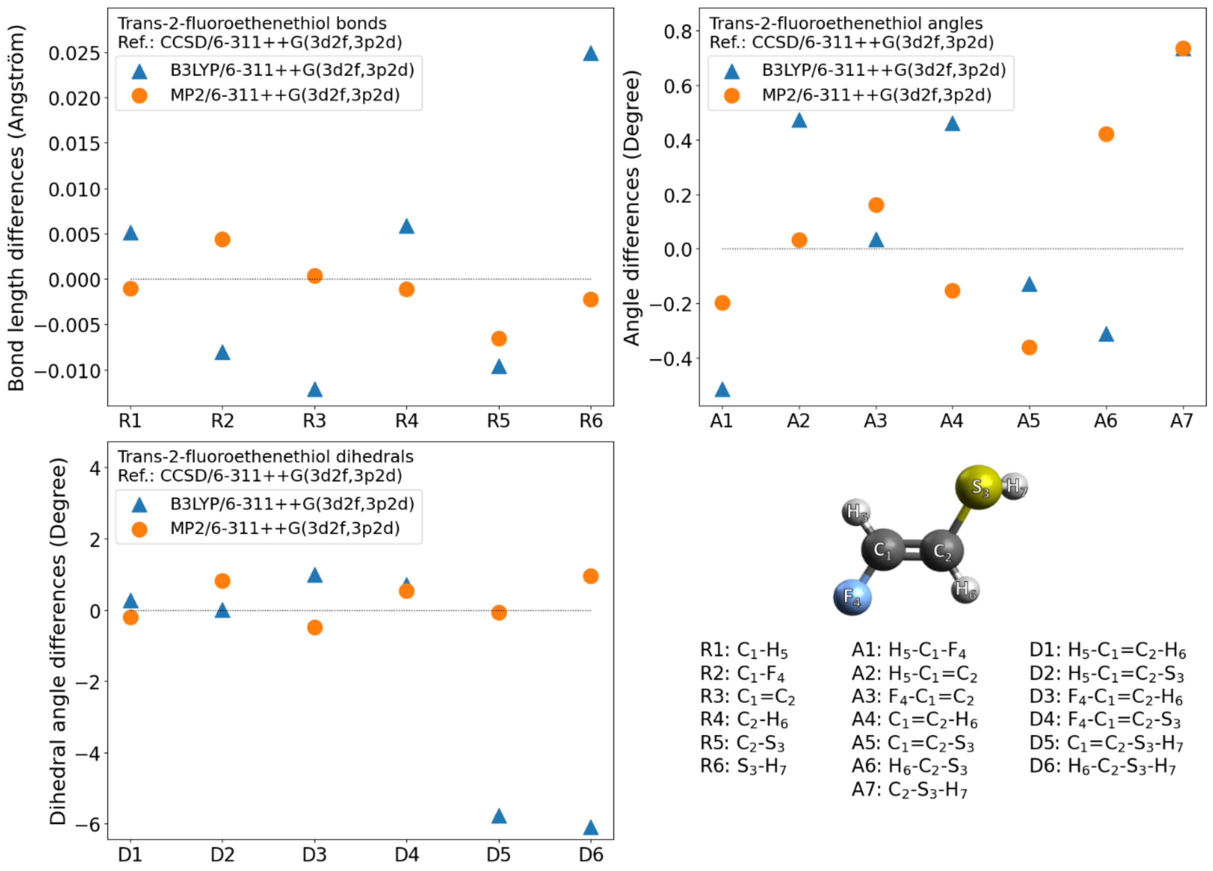

Figure S10. Top left: bond length differences between MP2/6-311++G(3d2f,3p2d) (Blue) and B3LYP/6-311++G(3d2f,3p2d) (Orange) with CCSD /6-311++G(3d2f,3p2d) level of theory in the case of Trans-2-fluoroethenethiol. Top right: angle differences between MP2/6-311++G(3d2f,3p2d) (Blue) and B3LYP/6-311++G(3d2f,3p2d) (Orange) with CCSD /6-311++G(3d2f,3p2d) level of theory in the case of Trans-2-fluoroethenethiol. Bottom left: dihedral angle differences between MP2/6-311++G(3d2f,3p2d) (Blue) and B3LYP/6-311++G(3d2f,3p2d) (Orange) with CCSD /6-311++G(3d2f,3p2d) level of theory in the case of Trans-2-fluoroethenethiol.

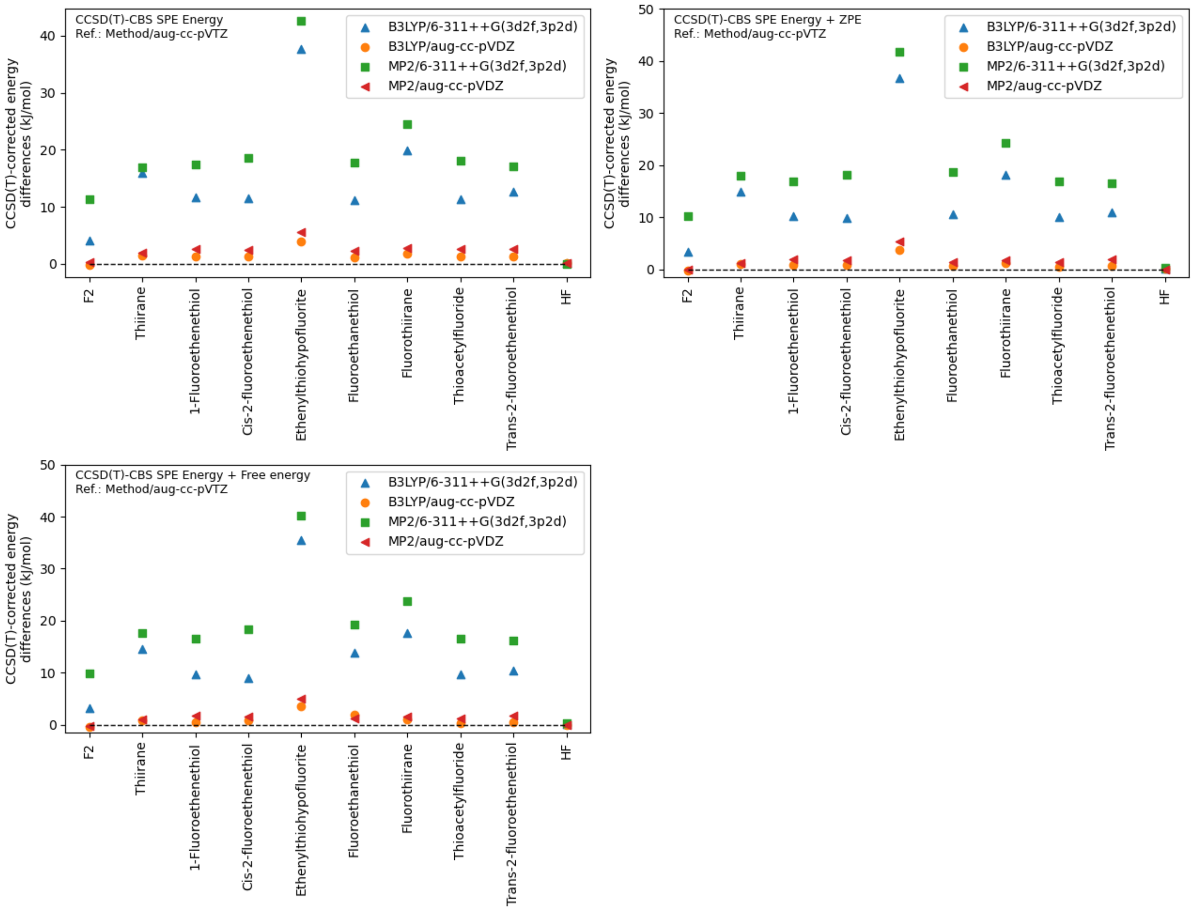

Figure S11. Top left: CCSD(T)-CBS/(aug-cc-pVTZ:aug-cc-pVQZ) single point energy (SPE) differences between (B3LYP or MP2)/6-311++G(3d2f,3p2d), (B3LYP or MP2)/aug-cc-pVDZ and (B3LYP or MP2)/aug-cc-pVTZ (reference) for every molecule. Top right: CCSD(T)-CBS/(aug-cc-pVTZ:aug-cc-pVQZ) single point energy (SPE) + Zero Point Energy (ZPE) differences between (B3LYP or MP2)/6-311++G(3d2f,3p2d), (B3LYP or MP2)/aug-cc-pVDZ and (B3LYP or MP2)/aug-cc-pVTZ (reference) for every molecule. Bottom left: CCSD(T)-CBS/(aug-cc-pVTZ:aug-cc-pVQZ) single point energy (SPE) + thermal free energy differences between (B3LYP or MP2)/6-311++G(3d2f,3p2d), (B3LYP or MP2)/aug-cc-pVDZ and (B3LYP or MP2)/aug-cc-pVTZ (reference) for every molecule. The geometry optimization has been performed at (B3LYP, MP2 or CCSD)/6-311++G(3d2f,3p2d) level in all cases.

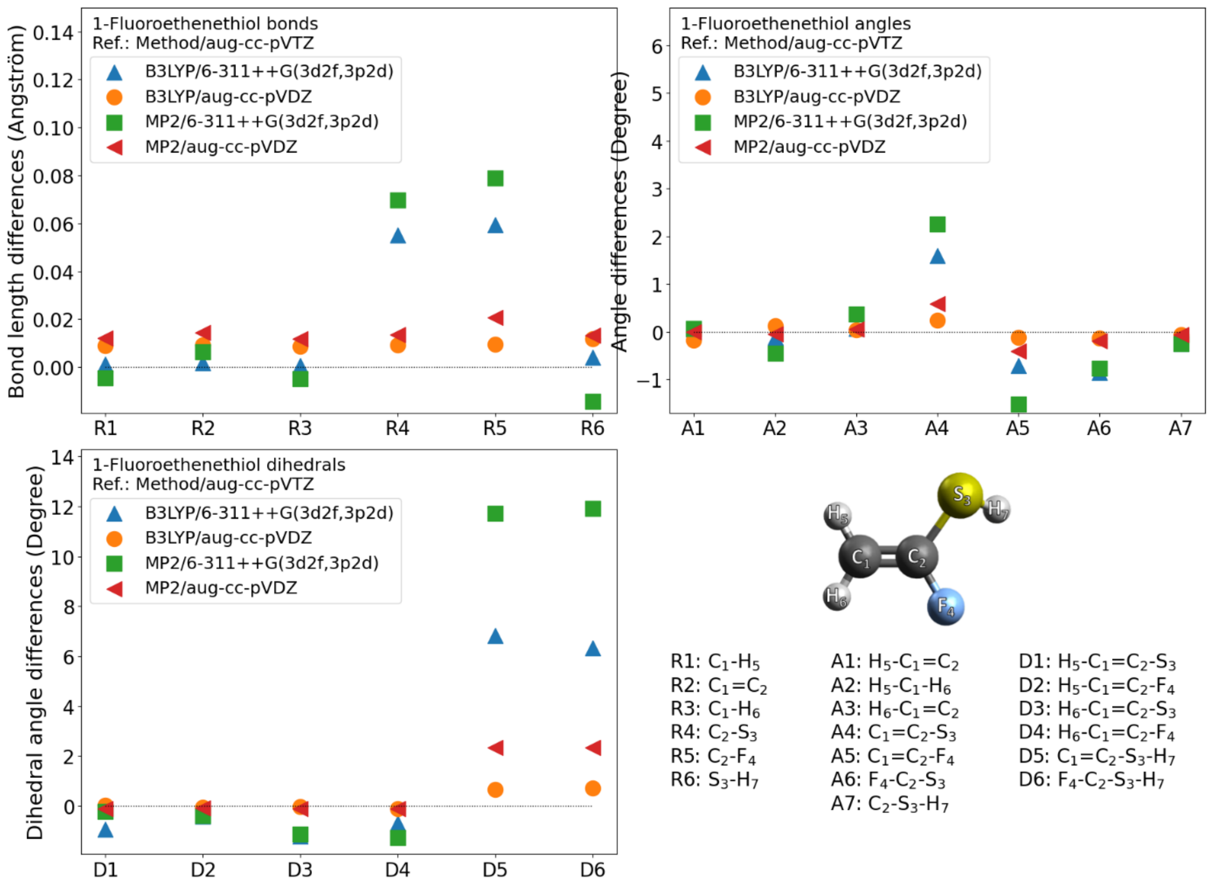

Figure S12. Top left: bond length differences between (B3LYP or MP2)/6-311++G(3d2f,3p2d) and (B3LYP or MP2)/aug-cc-pVDZ with (B3LYP or MP2)/aug-cc-pVTZ level of theory in the case of 1-Fluoroethenethiol. Top right: angle differences between (B3LYP or MP2)/6-311++G(3d2f,3p2d) and (B3LYP or MP2)/aug-cc-pVDZ with (B3LYP or MP2)/aug-cc-pVTZ level of theory in the case of 1-Fluoroethenethiol. Bottom left: dihedral angle differences between (B3LYP or MP2)/6-311++G(3d2f,3p2d) and (B3LYP or MP2)/aug-cc-pVDZ with (B3LYP or MP2)/aug-cc-pVTZ level of theory in the case of 1-Fluoroethenethiol.

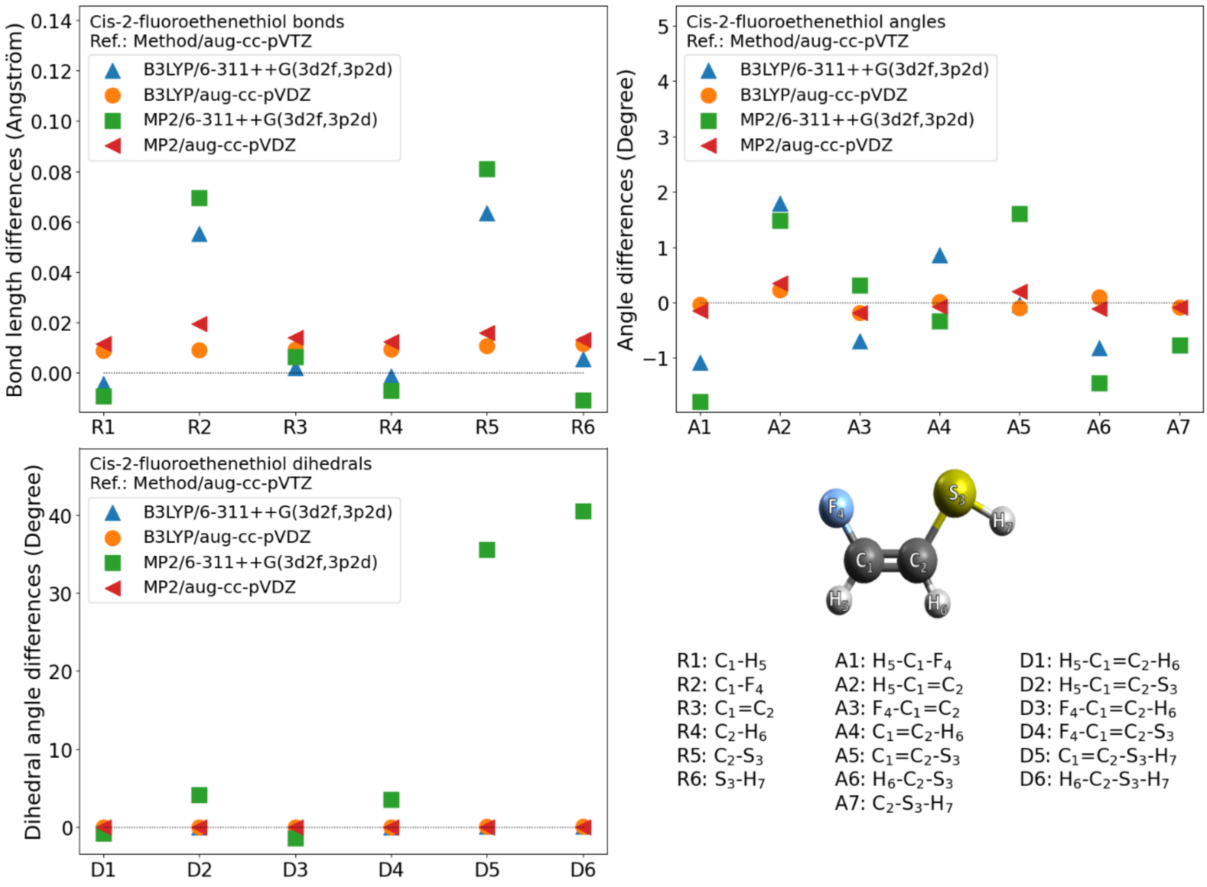

Figure S13. Top left: bond length differences between (B3LYP or MP2)/6-311++G(3d2f,3p2d) and (B3LYP or MP2)/aug-cc-pVDZ with (B3LYP or MP2)/aug-cc-pVTZ level of theory in the case of Cis-2-fluoroethenethiol. Top right: angle differences between (B3LYP or MP2)/6-311++G(3d2f,3p2d) and (B3LYP or MP2)/aug-cc-pVDZ with (B3LYP or MP2)/aug-cc-pVTZ level of theory in the case of Cis-2-fluoroethenethiol. Bottom left: dihedral angle differences between (B3LYP or MP2)/6-311++G(3d2f,3p2d) and (B3LYP or MP2)/aug-cc-pVDZ with (B3LYP or MP2)/aug-cc-pVTZ level of theory in the case of Cis-2-fluoroethenethiol.

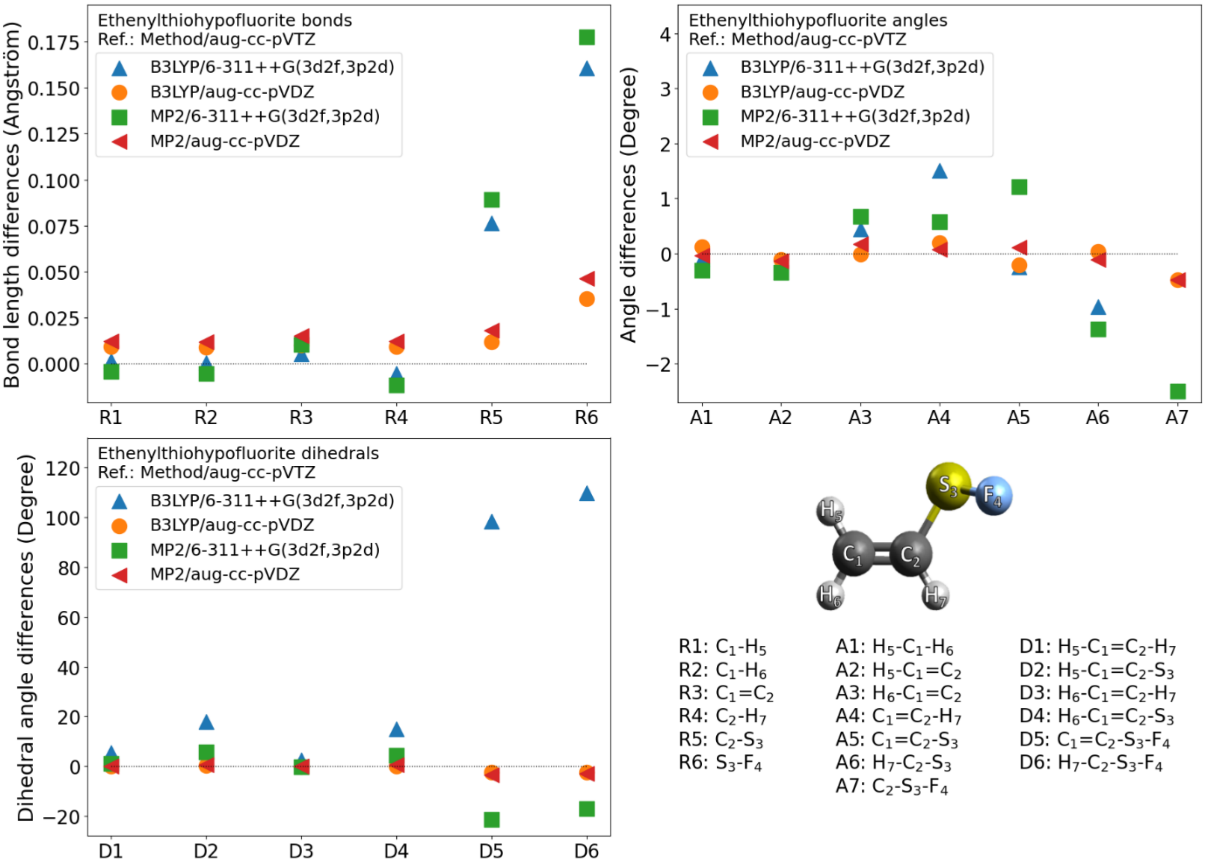

Figure S14. Top left: bond length differences between (B3LYP or MP2)/6-311++G(3d2f,3p2d) and (B3LYP or MP2)/aug-cc-pVDZ with (B3LYP or MP2)/aug-cc-pVTZ level of theory in the case of Ethenylthiohypofluorite. Top right: angle differences between (B3LYP or MP2)/6-311++G(3d2f,3p2d) and (B3LYP or MP2)/aug-cc-pVDZ with (B3LYP or MP2)/aug-cc-pVTZ level of theory in the case of Ethenylthiohypofluorite. Bottom left: dihedral angle differences between (B3LYP or MP2)/6-311++G(3d2f,3p2d) and (B3LYP or MP2)/aug-cc-pVDZ with (B3LYP or MP2)/aug-cc-pVTZ level of theory in the case of Ethenylthiohypofluorite.

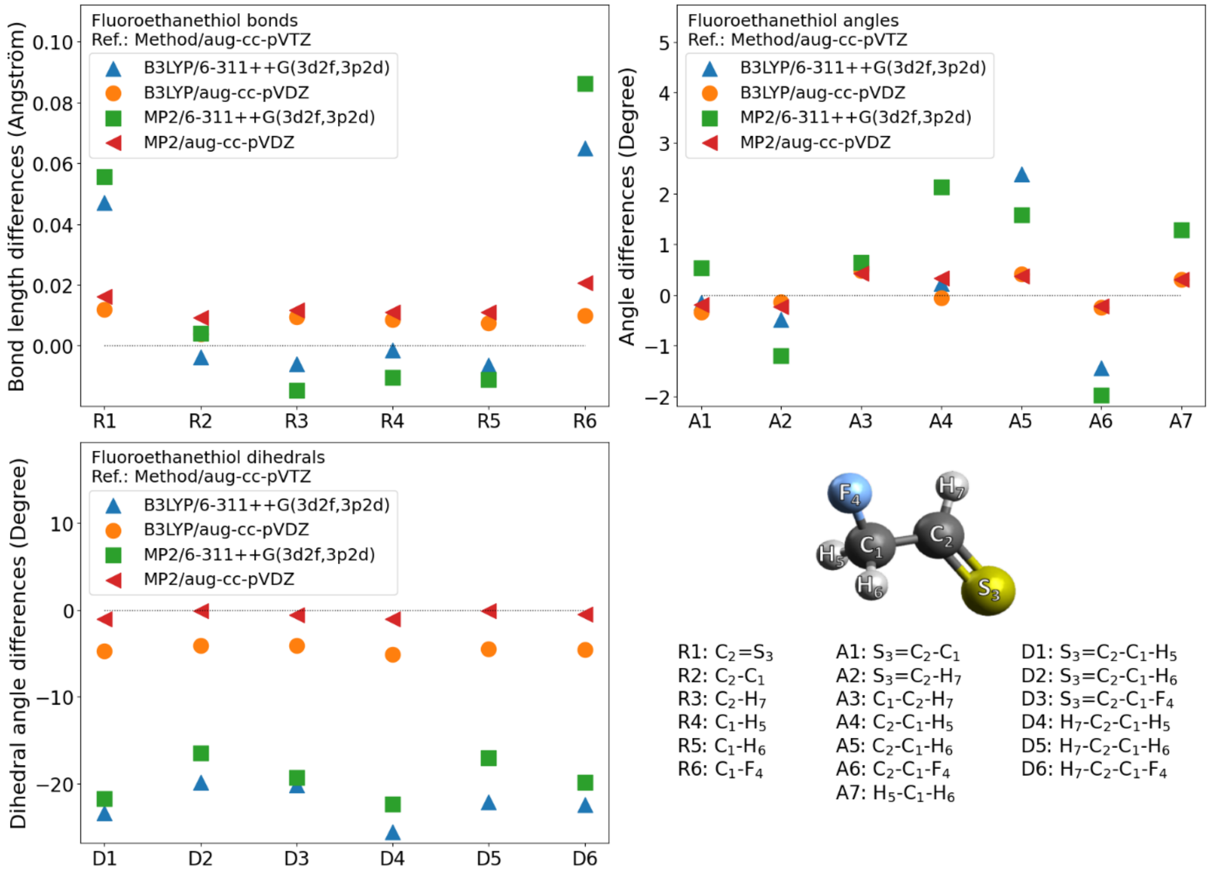

Figure S15. Top left: bond length differences between (B3LYP or MP2)/6-311++G(3d2f,3p2d) and (B3LYP or MP2)/aug-cc-pVDZ with (B3LYP or MP2)/aug-cc-pVTZ level of theory in the case of Fluoroethanethiol. Top right: angle differences between (B3LYP or MP2)/6-311++G(3d2f,3p2d) and (B3LYP or MP2)/aug-cc-pVDZ with (B3LYP or MP2)/aug-cc-pVTZ level of theory in the case of Fluoroethanethiol. Bottom left: dihedral angle differences between (B3LYP or MP2)/6-311++G(3d2f,3p2d) and (B3LYP or MP2)/aug-cc-pVDZ with (B3LYP or MP2)/aug-cc-pVTZ level of theory in the case of Fluoroethanethiol.

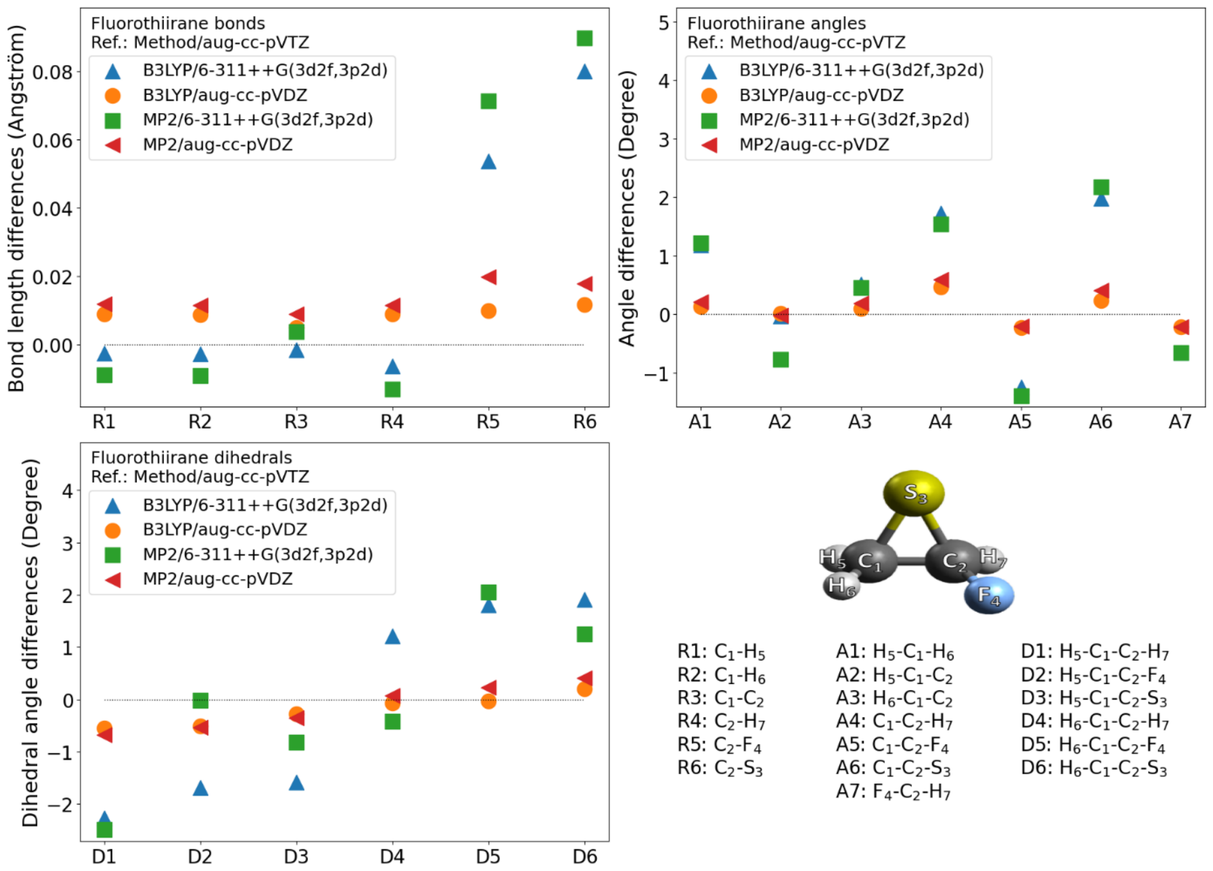

Figure S16. Top left: bond length differences between (B3LYP or MP2)/6-311++G(3d2f,3p2d) and (B3LYP or MP2)/aug-cc-pVDZ with (B3LYP or MP2)/aug-cc-pVTZ level of theory in the case of Fluorothiirane. Top right: angle differences between (B3LYP or MP2)/6-311++G(3d2f,3p2d) and (B3LYP or MP2)/aug-cc-pVDZ with (B3LYP or MP2)/aug-cc-pVTZ level of theory in the case of Fluorothiirane. Bottom left: dihedral angle differences between (B3LYP or MP2)/6-311++G(3d2f,3p2d) and (B3LYP or MP2)/aug-cc-pVDZ with (B3LYP or MP2)/aug-cc-pVTZ level of theory in the case of Fluorothiirane.

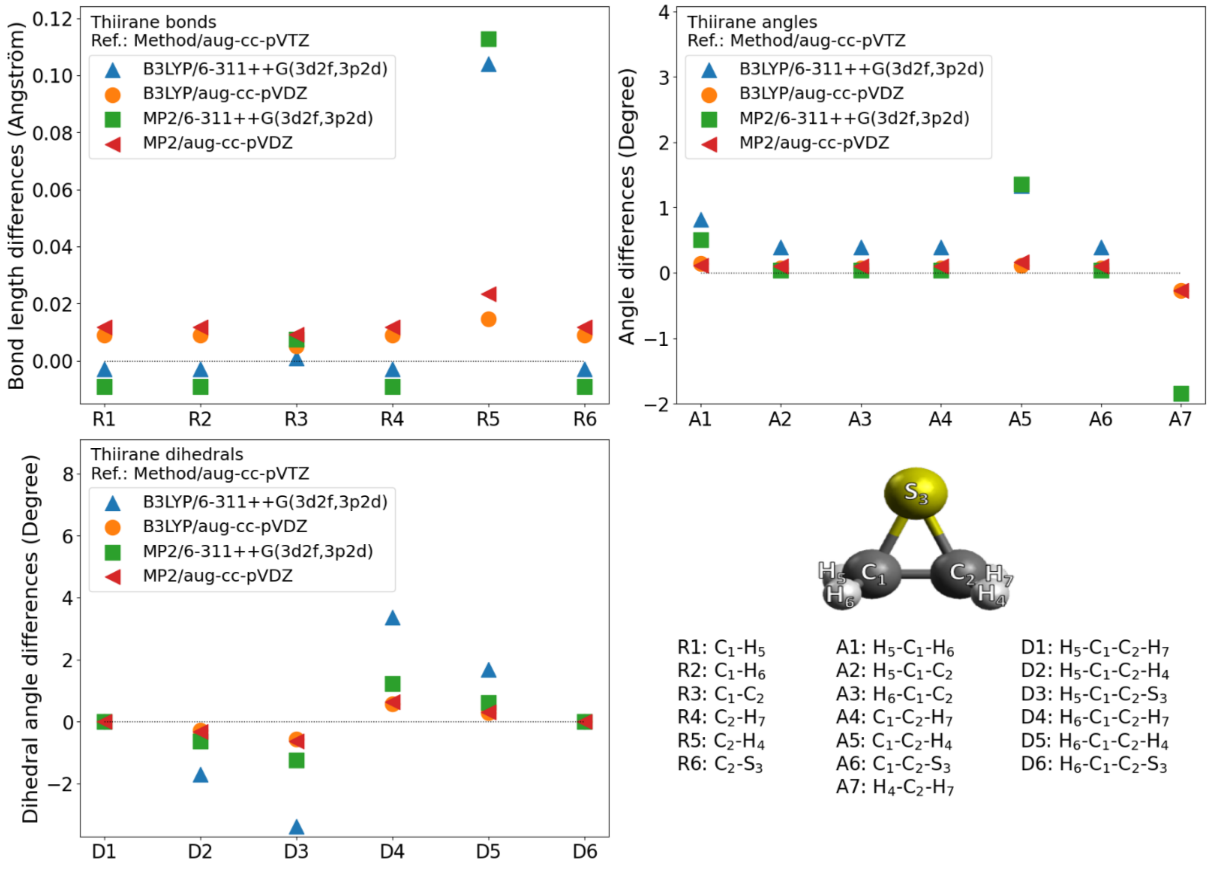

Figure S17. Top left: bond length differences between (B3LYP or MP2)/6-311++G(3d2f,3p2d) and (B3LYP or MP2)/aug-cc-pVDZ with (B3LYP or MP2)/aug-cc-pVTZ level of theory in the case of Thiirane. Top right: angle differences between (B3LYP or MP2)/6-311++G(3d2f,3p2d) and (B3LYP or MP2)/aug-cc-pVDZ with (B3LYP or MP2)/aug-cc-pVTZ level of theory in the case of Thiirane. Bottom left: dihedral angle differences between (B3LYP or MP2)/6-311++G(3d2f,3p2d) and (B3LYP or MP2)/aug-cc-pVDZ with (B3LYP or MP2)/aug-cc-pVTZ level of theory in the case of Thiirane.

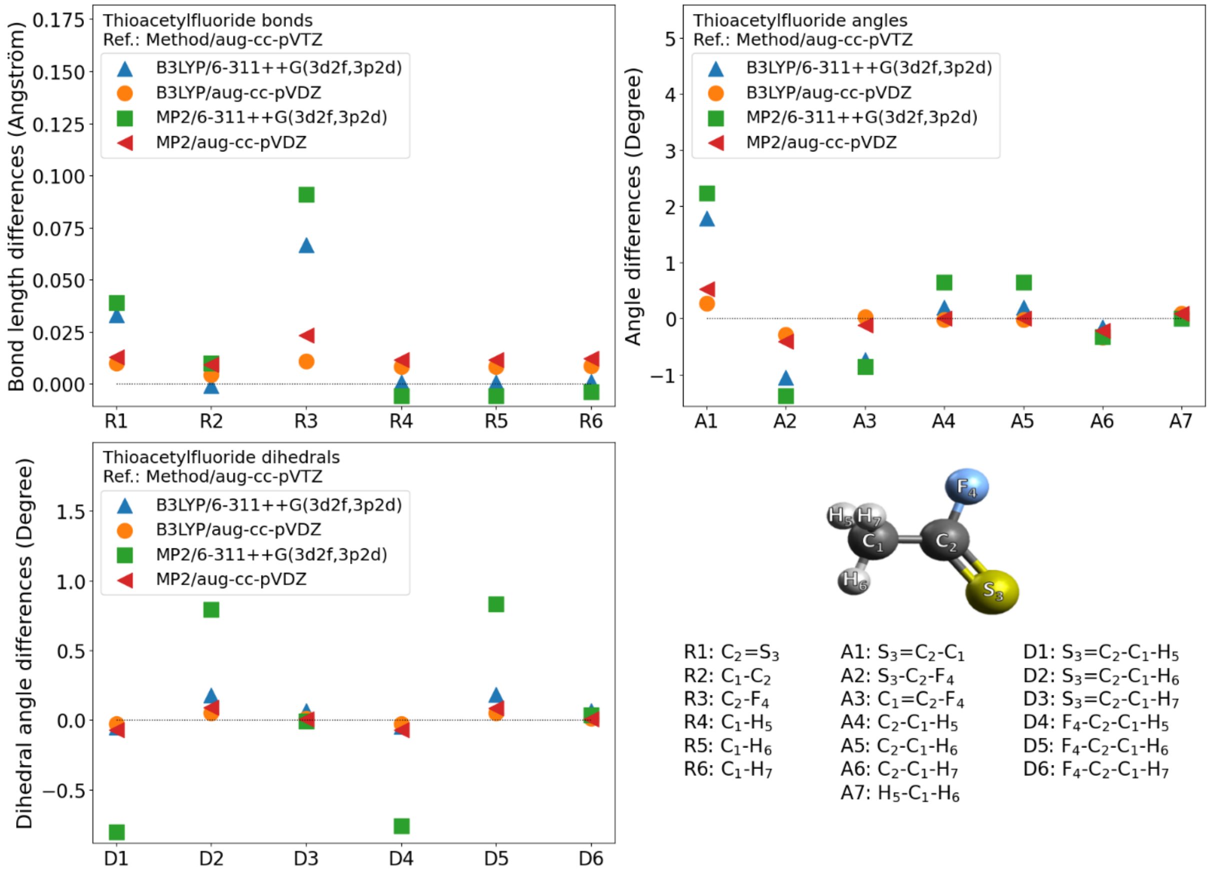

Figure S18. Top left: bond length differences between (B3LYP or MP2)/6-311++G(3d2f,3p2d) and (B3LYP or MP2)/aug-cc-pVDZ with (B3LYP or MP2)/aug-cc-pVTZ level of theory in the case of Thioacetylfluoride. Top right: angle differences between (B3LYP or MP2)/6-311++G(3d2f,3p2d) and (B3LYP or MP2)/aug-cc-pVDZ with (B3LYP or MP2)/aug-cc-pVTZ level of theory in the case of Thioacetylfluoride. Bottom left: dihedral angle differences between (B3LYP or MP2)/6-311++G(3d2f,3p2d) and (B3LYP or MP2)/aug-cc-pVDZ with (B3LYP or MP2)/aug-cc-pVTZ level of theory in the case of Thioacetylfluoride.

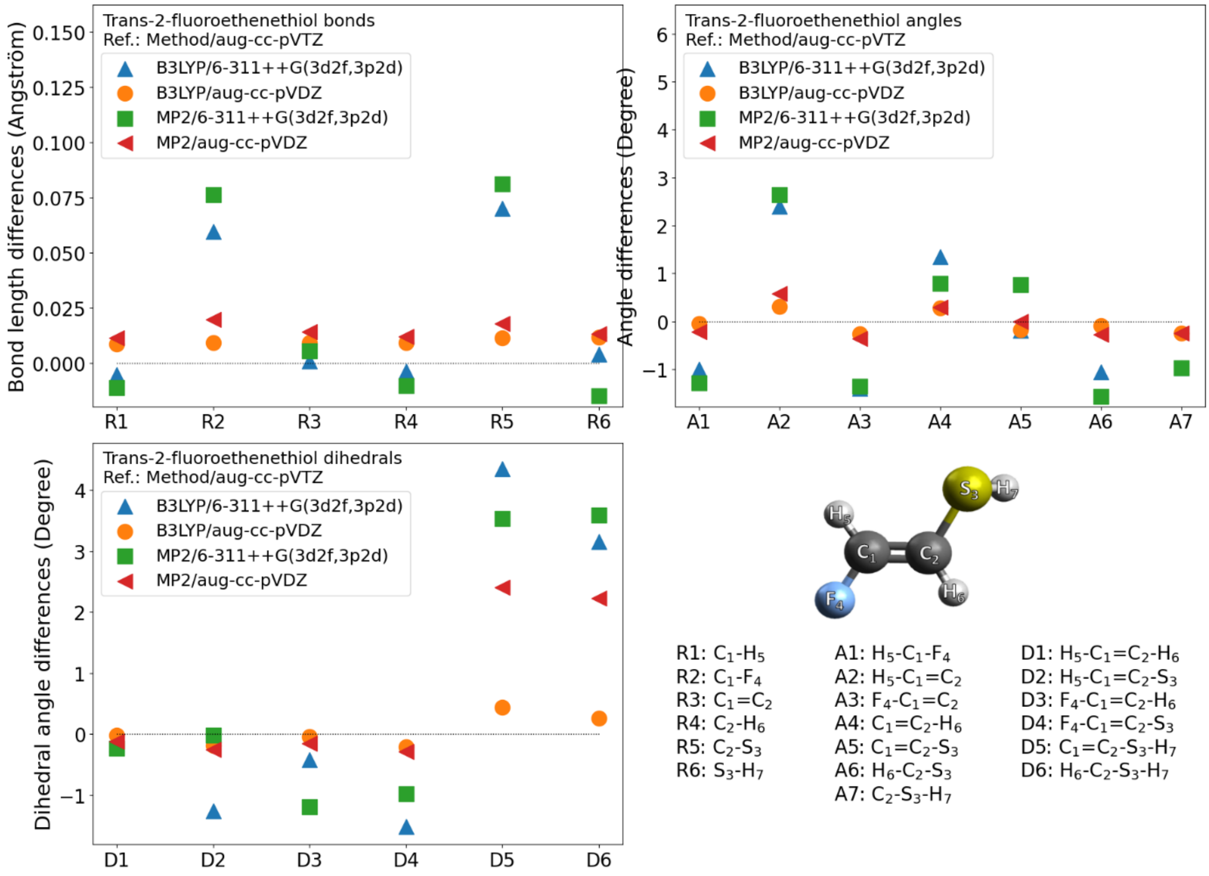

Figure S19. Top left: bond length differences between (B3LYP or MP2)/6-311++G(3d2f,3p2d) and (B3LYP or MP2)/aug-cc-pVDZ with (B3LYP or MP2)/aug-cc-pVTZ level of theory in the case of Trans-2-fluoroethenethiol. Top right: angle differences between (B3LYP or MP2)/6-311++G(3d2f,3p2d) and (B3LYP or MP2)/aug-cc-pVDZ with (B3LYP or MP2)/aug-cc-pVTZ level of theory in the case of Trans-2-fluoroethenethiol. Bottom left: dihedral angle differences between (B3LYP or MP2)/6-311++G(3d2f,3p2d) and (B3LYP or MP2)/aug-cc-pVDZ with (B3LYP or MP2)/aug-cc-pVTZ level of theory in the case of Trans-2-fluoroethenethiol.

Boltzmann distribution

In Figure 19, the Boltzmann relative distribution has been computed as function of temperature over range going from 100 K to 100000 K corresponding to an energy range of 0.83 kJ/mol to 831.43 kJ/mol. One can observe that if the thioacetylfluoride is dominating the distribution at low energy, it changes rapidly over ~10 kJ/mol. Over 100 kJ/mol, the relative ratio are between 10 and 20% for every compounds.

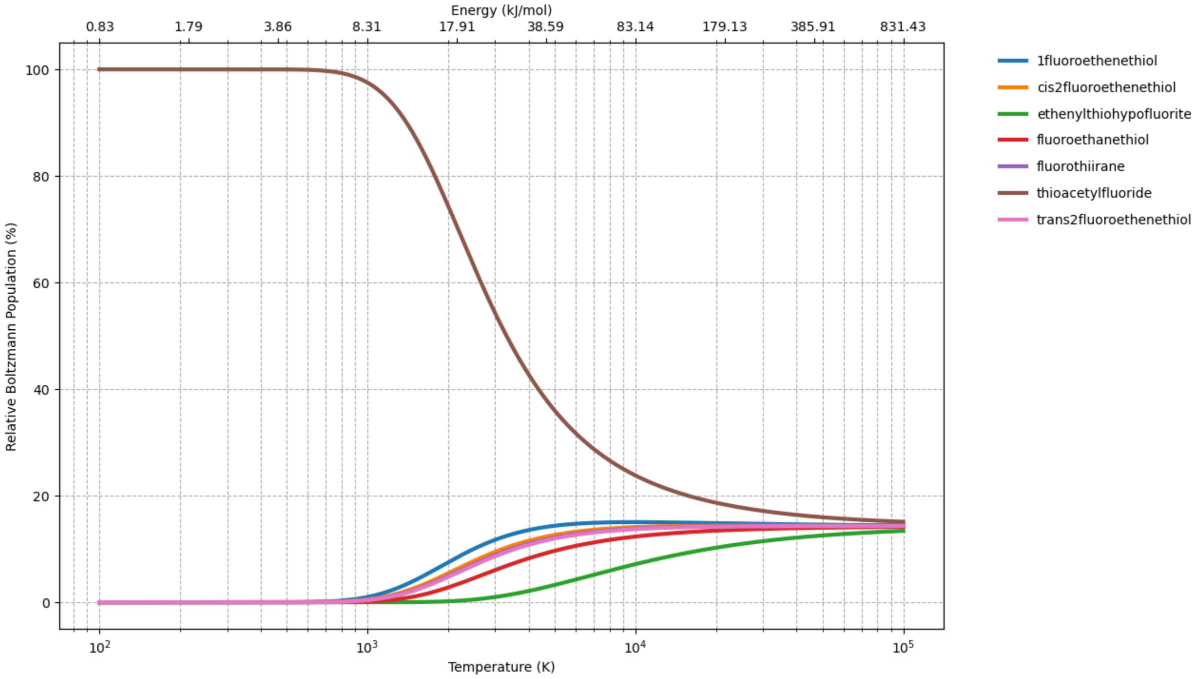

Figure S20. Evolution of the Boltzmann relative distribution of the products of the reaction Thiirane + F_2 as a function of temperature. The corresponding energy (in kJ/mol) is on the upper axis.

Optimized Geometry

Below are listed the optimized geometry at MP2/aug-cc-pVTZ level

- 1-fluoroethenethiol

S	-1.2903230000	0.1550120000	-0.0612490000
F	0.9229400000	-1.2377070000	-0.0338840000
C	0.4597470000	0.0269450000	0.0184800000
C	1.3031290000	1.0555640000	0.0363200000
H	2.3653660000	0.8839030000	-0.0441530000
H	-1.5199020000	-0.7760040000	0.8743530000
H	0.9159780000	2.0562270000	0.1259480000

- Cis-2-fluoroethenethiol

S	1.3314620000	-0.3214670000	-0.0000320000
F	-1.6198550000	-0.7690670000	0.0000290000
C	0.0379090000	0.8684220000	-0.0000070000
C	-1.2470380000	0.5244760000	-0.0000050000
H	2.3204460000	0.5756190000	0.0004330000
H	0.2858930000	1.9178990000	-0.0000610000
H	-2.0762730000	1.2141630000	-0.0000400000

- Ethenylthiohypofluorite

S	-0.5337120000	0.5661310000	0.1372040000
F	-1.6437190000	-0.5757750000	-0.2651470000
C	0.8759000000	-0.4387150000	0.2792570000
C	2.0392450000	-0.0349620000	-0.2485800000
H	0.7978590000	-1.3127200000	0.9142000000
H	2.0859010000	0.8333150000	-0.8911940000
H	2.9582280000	-0.5546400000	-0.0160130000

- F_2

F	0.0000000000	0.0000000000	-0.7006890000
F	0.0000000000	0.0000000000	0.7006890000

- Fluoroethanethiol

S	1.6845210000	-0.0495730000	0.0582620000
F	-2.1847620000	0.2122980000	0.1430590000
C	-1.0176770000	-0.4958070000	-0.1135040000
C	0.1547140000	0.4353290000	-0.1306920000
H	-0.0953760000	1.4798970000	-0.3134030000
H	-0.8954170000	-1.2623410000	0.6497370000
H	-1.1209150000	-0.9722060000	-1.0908920000

- Fluorothiirane

S	-1.0661570000	-0.4645940000	-0.0562860000
F	1.6384290000	-0.3797650000	-0.2368910000
C	0.5672880000	0.0738320000	0.4733420000

C	-0.2175590000	1.1617430000	-0.1241010000
H	0.7625730000	0.0429570000	1.5360490000
H	0.0772970000	1.4667550000	-1.1184400000
H	-0.6255860000	1.9282350000	0.5195320000

- HF

F	0.0000000000	0.0000000000	0.0921840000
H	0.0000000000	0.0000000000	-0.8296520000

- Thiirane

S	0.8653050000	0.0000030000	0.0000000000
C	-0.7984210000	0.7399470000	0.0000000000
C	-0.7984120000	-0.7399530000	0.0000000000
H	-1.0659820000	1.2470000000	-0.9159090000
H	-1.0659820000	1.2470000000	0.9159090000
H	-1.0659610000	-1.2470070000	-0.9159120000
H	-1.0659610000	-1.2470070000	0.9159120000

- Thioacetylfluoride

S	1.3406900000	-0.1673870000	0.0000000000
F	-0.7760870000	1.2963740000	-0.0000030000
C	-1.3460140000	-0.9554910000	-0.0000080000
C	-0.2507030000	0.0510860000	0.0000080000
H	-0.9418960000	-1.9609920000	-0.0009100000
H	-1.9725570000	-0.8002830000	-0.8789820000
H	-1.9714930000	-0.8014640000	0.8799280000

- Trans-2-fluoroethenethiol

S	1.6321570000	-0.1192600000	-0.0758520000
F	-2.3055670000	0.0516070000	-0.0164700000
C	-0.0191370000	0.5004620000	-0.0136190000
C	-1.0302490000	-0.3611590000	0.0608640000
H	2.0720290000	0.4808380000	1.0377390000
H	-0.2066070000	1.5580070000	-0.1355370000
H	-0.9335200000	-1.4309640000	0.1761890000

Gaussian inputs

- 1-fluoroethenethiol

```
%chk=1fluoroethenethiol.MP2.aug-cc-pVTZ.chk
%mem=1GW
%Nproc=4
# opt=calcall freq=noraman MP2/aug-cc-pVTZ

1fluoroethenethiol

0 1
 C
 H      1    B1
 C      1    B2   2    A1
 S      3    B3   1    A2   2    D1   0
 H      4    B4   3    A3   1    D2   0
 H      1    B5   3    A4   4    D3   0
 F      3    B6   1    A5   4    D4   0

   B1       1.07000000
   B2       1.35520000
   B3       1.78000000
   B4       1.31000000
   B5       1.07000000
   B6       1.35000000
   A1     119.88652694
   A2     119.88652694
   A3     109.50000006
   A4     120.22694612
   A5     120.22694612
   D1       0.00000000
   D2    -168.73540252
   D3    -180.00000000
   D4    -180.00000000
```

- Cis-2-fluoroethenethiol

```
%chk=cis2fluoroethenethiol.MP2.aug-cc-pVTZ.chk
%mem=1GW
%Nproc=4
# opt=calcall freq=noraman MP2/aug-cc-pVTZ

Cis-2-fluoroethenethiol

0 1
 C
 H      1    B1
 F      1    B2   2    A1
 C      1    B3   2    A2   3    D1   0
 H      4    B4   1    A3   2    D2   0
 S      4    B5   1    A4   2    D3   0
 H      6    B6   4    A5   1    D4   0
```

```
  B1        1.07000000
  B2        1.35000000
  B3        1.35520000
  B4        1.07000000
  B5        1.78000000
  B6        1.31000000
  A1      119.88652694
  A2      119.88652694
  A3      120.22694612
  A4      119.88652694
  A5      109.50000006
  D1     -180.00000000
  D2      -35.53013159
  D3      144.46986841
  D4     -168.73540252
```

- Ethenylhypofluorite

```
%chk=ethenylthiohypofluorite.MP2.aug-cc-pVTZ.chk
%mem=1GW
%Nproc=4
# opt=calcall freq=noraman MP2/aug-cc-pVTZ

Ethenylthiohypofluorite

0 1
 C
 H     1    B1
 H     1    B2   2    A1
 C     1    B3   3    A2   2    D1   0
 H     4    B4   1    A3   3    D2   0
 S     4    B5   1    A4   3    D3   0
 F     6    B6   4    A5   1    D4   0

  B1        1.07000000
  B2        1.07000000
  B3        1.35520000
  B4        1.07000000
  B5        1.78000000
  B6        1.59000000
  A1      119.88652694
  A2      119.88652694
  A3      119.88652694
  A4      120.22694612
  A5      109.50000006
  D1     -180.00000000
  D2      180.00000000
  D3       -0.00000000
  D4      180.00000000
```

- Fluoroethanethiol

```
%chk=fluoroethanethiol.MP2.aug-cc-pVTZ.chk
%mem=1GW
```

```
%Nproc=4
# opt=calcall freq=noraman MP2/aug-cc-pVTZ

Fluoroethanethiol

0 1
 C
 S     1   B1
 C     1   B2   2    A1
 H     3   B3   1    A2   2    D1   0
 H     3   B4   1    A3   2    D2   0
 F     3   B5   1    A4   2    D3   0
 H     1   B6   3    A5   2    D4   0

   B1      1.56640000
   B2      1.54000000
   B3      1.07000000
   B4      1.07000000
   B5      1.35000000
   B6      1.07000000
   A1    119.88652694
   A2    109.47120255
   A3    109.47120255
   A4    109.47123134
   A5    119.88652694
   D1    -59.99999637
   D2     60.00001842
   D3   -180.00000000
   D4   -180.00000000
```

- Fluorothiirane

```
%chk=fluorothiirane.MP2.aug-cc-pVTZ.chk
%mem=1GW
%nproc=4
# opt=calcall freq=noraman MP2/aug-cc-pVTZ

Fluorothiirane

0 1
 C
 H     1   B1
 H     1   B2   2    A1
 C     1   B3   2    A2   3    D1   0
 H     4   B4   1    A3   2    D2   0
 F     4   B5   1    A4   2    D3   0
 S     4   B6   1    A5   2    D4   0

   B1      1.07000000
   B2      1.07000000
   B3      1.77423324
   B4      1.07000000
   B5      1.35000000
   B6      2.01324228
```

```
  A1         109.47123134
  A2         105.91884412
  A3         118.29531290
  A4         118.29533251
  A5          70.47120005
  D1         113.76158415
  D2           9.80441709
  D3        -126.22905142
  D4         121.78770000
```

- Thiirane

```
%chk=thiirane.MP2.aug-cc-pVTZ.chk
%mem=1GW
%Nproc=4
# opt=calcall freq=noraman MP2/aug-cc-pVTZ

Thiirane

0 1
 C
 H      1    B1
 H      1    B2   2    A1
 C      1    B3   2    A2   3    D1   0
 H      4    B4   1    A3   2    D2   0
 S      4    B5   1    A4   2    D3   0
 H      4    B6   1    A5   6    D4   0

  B1          1.07000000
  B2          1.07000000
  B3          1.77423324
  B4          1.07000000
  B5          2.01324228
  B6          1.07000000
  A1        109.47123134
  A2        105.91884412
  A3        118.29531290
  A4         70.47120005
  A5        118.29533251
  D1        113.76158415
  D2          9.80441709
  D3        121.78770000
  D4        111.98324858
```

- Thioacetylfluoride

```
%chk=thioacetylfluoride.MP2.aug-cc-pVTZ.chk
%mem=1GW
%Nproc=4
# opt=calcall freq=noraman MP2/aug-cc-pVTZ

Thioacetylfluoride

0 1
```

```
S
C    1   B1
C    2   B2  1   A1
H    3   B3  2   A2  1   D1  0
H    3   B4  2   A3  1   D2  0
H    3   B5  2   A4  1   D3  0
F    2   B6  1   A5  3   D4  0

  B1      1.56640000
  B2      1.54000000
  B3      1.07000000
  B4      1.07000000
  B5      1.07000000
  B6      1.35000000
  A1    119.88652694
  A2    109.47120255
  A3    109.47120255
  A4    109.47123134
  A5    120.22694612
  D1     60.00007749
  D2   -180.00000000
  D3    -59.99991511
  D4   -180.00000000
```

- Trans-2-fluoroethenethiol

```
%chk=trans2fluoroethenethiol.MP2.aug-cc-pVTZ.chk
%mem=1GW
%Nproc=4
# opt=calcall freq=noraman MP2/aug-cc-pVTZ

Trans-2-fluoroethenethiol

0 1
C
H    1   B1
F    1   B2  2   A1
C    1   B3  2   A2  3   D1  0
H    4   B4  1   A3  2   D2  0
S    4   B5  1   A4  2   D3  0
H    6   B6  4   A5  1   D4  0

  B1      1.07000000
  B2      1.35000000
  B3      1.35520000
  B4      1.07000000
  B5      1.78000000
  B6      1.31000000
  A1    119.88652694
  A2    119.88652694
  A3    120.22694612
  A4    119.88652694
  A5    109.50000006
  D1   -180.00000000
```

```
  D2        -180.00000000
  D3           0.00000000
  D4        -168.73540252
```

- F2

```
%chk=F2.MP2.aug-cc-pVTZ.chk
%mem=1GW
%Nproc=4
# opt=calcall freq=noraman MP2/aug-cc-pVTZ

F2

0 1
 F
 F            1       B1

  B1        1.07000000
```

- HF

```
%chk=HF.MP2.aug-cc-pVTZ.chk
%mem=1GW
%Nproc=4
# opt=calcall freq=noraman MP2/aug-cc-pVTZ

HF

0 1
 F
 H            1       B1

  B1        1.07000000
```

Molpro input

```
memory, 400, m; ! in mWords per processor
 file, 1, molecule.int, new;
 file, 2, molecule.wfu, new;

gprint, basis;

geomtyp=xyz;
geometry={
include, molecule.xyz;
}

spin=0
charge=0

$l_basis = [aug-cc-pVTZ, aug-cc-pVQZ]

! CCSD(T) calculation with VTZ, VQZ basis sets
do k=1,#i_basis
   basis={
   default=$l_basis(k);
   }

   {hf;}
   {ccsd(t)};

   eref_x(k) = energr;
   ecor_x(k) = energy-energr;

enddo;

! Extrapolation with avtz, avqz basis sets with 2 points
! For HF energy: A. Karton and J. M. L. Martin, Theor. Chem. Acc. 115, 330 (2006)
! For corr. energy by LH3:

extrapolate, basis=avtz:avqz, eref=eref_x, ecorr=ecor_x, method_r=km, npc=2;
ecbs = energy(3);
```
